# Unveiling *Chlamydia trachomatis* immunity in urogenital secretions: A systematic review

**DOI:** 10.1016/j.isci.2025.113453

**Published:** 2025-08-28

**Authors:** Anne Van Caesbroeck, Marijana Lipovac, Eef van den Borst, Paolo Palma, Laura Téblick, Alex Vorsters

**Affiliations:** 1 Vaccinopolis, Centre for the Evaluation of Vaccination (CEV), Vaccine & Infectious Disease Institute (VAXINFECTIO), Faculty of Medicine and Health Sciences, University of Antwerp, 2610 Wilrijk-Antwerp, Belgium; 2 Centre of Medical Genetics, Faculty of Medicine and Health Sciences, University of Antwerp and Antwerp University Hospital, 2650 Edegem, Belgium; 3 Chair of Pediatrics, Department of Systems Medicine, Tor Vergata School of Medicine and Surgery, University of Rome Tor Vergata, 00133 Rome, Italy

**Keywords:** Sexual medicine, Mucosal Immunity, Sampling

## Abstract

*Chlamydia trachomatis* (CT) is a highly prevalent bacterial sexually transmitted infection (STI), associated with severe disease complications. CT targets a unique immunological environment: the genital tract epithelium. Although sampling the genital tract is challenging, previous studies have shown that genital CT-specific antibodies exhibit enhanced neutralizing capacity compared with serum antibodies. Furthermore, tissue-resident memory T (TRM) cells provide superior protection compared with circulating T cells. However, further research is required to identify correlates of protection and explore correlations between local and systemic responses. This review provides an overview of the sampling methods suitable for identifying mucosal immune biomarkers associated with CT infection, as well as the immunoassays used. We identified the microbiome, presence of coinfections, hormonal influences, genetics, and CT infection state, load, and genotype as confounding factors to be considered in trial design. Finally, we discuss challenges related to the detection of mucosal immune biomarkers and offer recommendations for future research.

## Highlights

• Genital samples are a source of DNA and humoral and cellular immune biomarkers
• Harmonization of sampling methods is essential
• No validated immunoassays for urogenital samples are available

## Graphical abstract

**Figure:**
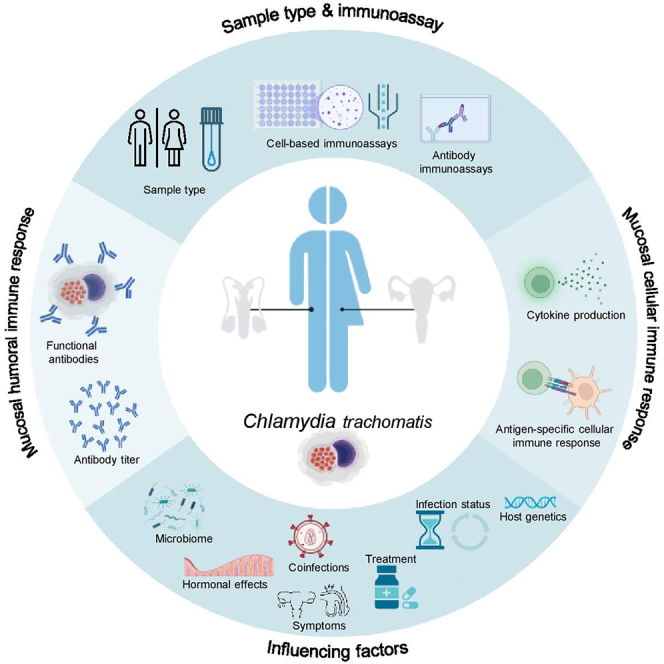

## Introduction

Genital *Chlamydia trachomatis* (CT) is a highly prevalent sexually transmitted infection (STI), with a global prevalence in 2020 estimated at 4.0% in women and 2.5% in men aged 15 to 49 years, affecting 69.9 million women and 58.6 million men.^1^^,^^2^^,^^3^ This represents a substantial economic and social burden.^4^ Despite being curable^5^ and preventable through consistent and correct use of barrier contraception methods,^6^^,^^7^ CT incidence continues to rise annually.^1^^,^^8^ The true scale of this epidemic is presumably underestimated, as CT is asymptomatic in over 70% of female and 50% of male infections.^9^^,^^10^^,^^11^ Moreover, opportunistic testing is largely limited to high-income countries^12^ and depends on national guidelines.^13^ Urogenital symptoms and sequelae can be severe, ranging from cervicitis and aberrant vaginal discharge to salpingitis, pelvic inflammatory disease, tubal factor infertility, ectopic pregnancy, and chronic pelvic pain in women and urethritis and epididymitis in men.^9^^,^^14^ It is clear that current screening and treatment programs are insufficient to contain this epidemic, highlighting the need for an effective preventive vaccine.

In STI research, a wide variety of sample types have been evaluated for collecting genital secretions. For women, these include cervical cytobrushes, cervicovaginal swabs, cervicovaginal lavage (CVL), first-void urine (FVU), menstrual cups, sponges, filter paper strips, and aspirate samples.^15^^,^^16^^,^^17^^,^^18^^,^^19^^,^^20^^,^^21^^,^^22^^,^^23^ For men, commonly used sample types include total ejaculate, seminal plasma, urine, expressed prostatic secretions (EPSs), and urethral swabs.^24^^,^^25^^,^^26^^,^^27^ Studies monitoring the impact of future STI vaccines could benefit from incorporating standardized site-specific biological samples. Mucosal samples are versatile, as most can be used for both DNA testing and immunological endpoints,^28^ supporting their use as a universal sample in vaccine research. Sample types such as vaginal swabs and urine are particularly advantageous because they can be collected at home, a factor that has been shown to improve participant acceptance and compliance in clinical trials.^29^^,^^30^^,^^31^ Additionally, site-specific samples provide insights on local antibodies and immune cells, which show distinct differences compared with systemic immune responses in terms of both abundance and functionality.^32^^,^^33^^,^^34^^,^^35^^,^^36^^,^^37^ The ideal sample type for evaluating CT mucosal immune responses in genital secretions has yet to be determined. It is evident that not all samples are universally suitable for every biomarker or immunoassay, underscoring a significant research gap in this area.

Hence, the objective of this review is to give a comprehensive overview of local mucosal immune biomarkers associated with CT infection and explore their association with systemic immune responses. Identified prognostic biomarkers may support future vaccine monitoring studies, since a correlate of protection is unknown. This review will discuss methods used for sampling the local urogenital environment with regard to identification of those biomarkers and outline potential confounding factors influencing the detection and quantification of CT-associated immune responses. Finally, different immunoassays used for monitoring local immune responses will be discussed.

## Results

### Study selection and characteristics

**Figure 1:**
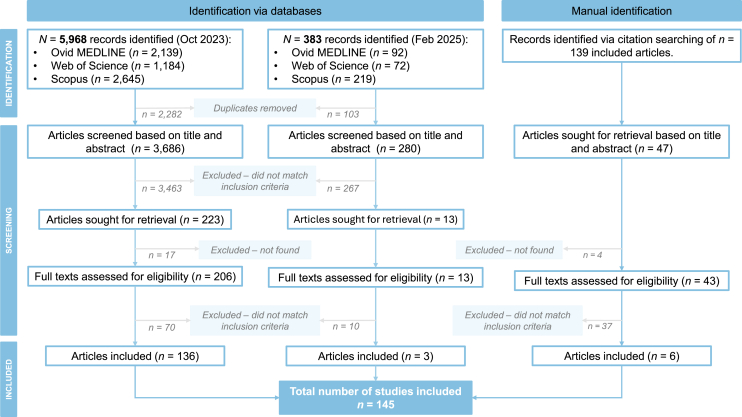
PRISMA flow diagram of the literature search Records were identified through three databases (Ovid MEDLINE, Web of Science, and Scopus). After duplicate removal, records were screened by title and abstract and excluded if they did not match the inclusion criteria. Articles were then retrieved for full-text screening and included if they matched inclusion criteria. Reference lists of included articles were manually screened for further records that were not yet included through database search and screened based on title and abstract. Full texts were included when appropriate.

A total of 5,968 records were identified through database searches conducted in October 2023 (Figure 1). Duplicates were removed using EndNote. Consequently, 3,686 articles remained for screening. An additional 47 articles were identified through screening of reference lists and evaluated based on title and abstract. Of those, 43 were retrieved and screened for eligibility. A total of 142 articles were included after full-text screening. An updated search in February 2025 identified an additional 383 records, of which three were included in the final analysis.

Of the 145 included publications, 85 reported on the detection of CT immune responses in female urogenital secretions, 54 in male urogenital secretions, and six included both male and female participants. The selected reports included cross-sectional studies (*n* = 81), case-control studies (*n* = 39), and cohort studies (*n* = 19). Remaining publication types comprised randomized controlled trials (*n* = 2), case reports and case series (*n* = 3), and letters to the editor (*n* = 1). In most studies, concerns were raised about the risk of bias. Individual risk of bias assessment scores are presented in Table S2. Meta-analysis was not performed due to data heterogeneity; instead, a narrative synthesis was conducted.

### Urogenital secretions used for monitoring local immune responses

**Figure 2:**
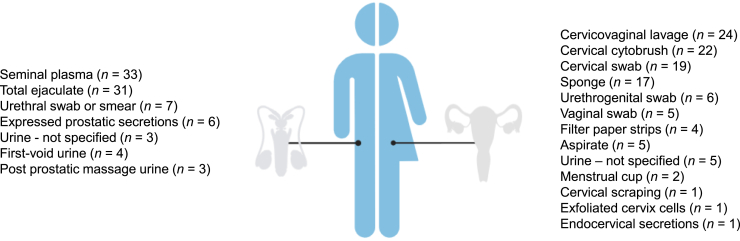
Sample types for collection of urogenital secretions in men and women Overview of sample types used in men (left) and women (right), in order of appearance. The number of studies using a certain sample type is noted in brackets.

An overview of specific characteristics and applications of the various sample types is provided in Table 1. Female urogenital secretions were collected using the following methods: CVL,^36^^,^^38^^,^^39^^,^^40^^,^^41^^,^^42^^,^^43^^,^^44^^,^^45^^,^^46^^,^^47^^,^^48^^,^^49^^,^^50^^,^^51^^,^^52^^,^^53^^,^^54^^,^^55^^,^^56^^,^^57^^,^^58^^,^^59^^,^^60^ cervical cytobrush,^37^^,^^39^^,^^41^^,^^42^^,^^43^^,^^44^^,^^45^^,^^50^^,^^51^^,^^54^^,^^60^^,^^61^^,^^62^^,^^63^^,^^64^^,^^65^^,^^66^^,^^67^^,^^68^^,^^69^^,^^70^^,^^71^ cervical swab,^57^^,^^69^^,^^72^^,^^73^^,^^74^^,^^75^^,^^76^^,^^77^^,^^78^^,^^79^^,^^80^^,^^81^^,^^82^^,^^83^^,^^84^^,^^85^^,^^86^^,^^87^^,^^88^ sponge,^37^^,^^49^^,^^66^^,^^89^^,^^90^^,^^91^^,^^92^^,^^93^^,^^94^^,^^95^^,^^96^^,^^97^^,^^98^^,^^99^^,^^100^^,^^101^^,^^102^ urethrogenital swabs,^107^^,^^108^^,^^109^^,^^110^^,^^111^^,^^112^ vaginal swabs,^93^^,^^103^^,^^104^^,^^105^^,^^106^ vaginal filter paper strips,^113^^,^^114^^,^^115^^,^^116^ aspirate,^63^^,^^114^^,^^117^^,^^118^^,^^119^ menstrual cup,^120^^,^^121^ urine,^72^^,^^75^^,^^176^^,^^177^^,^^178^ and cervical scraping^54^ (Figure 2). Notably, some sample collection methods were not clearly described, including the use of exfoliated cervical cells,^179^ endocervical secretions,^180^ or urine. Certain sample types were exclusively used for measuring soluble immune mediators (both cellular and humoral) including cervical swabs, sponges, filter paper strips, cervical aspirate, and menstrual cups. Urethrogenital swabs were used solely for antibody detection, whereas cervical scraping was used exclusively for phenotypical characterization of cells. Cervical cytobrushes, CVL, and vaginal swabs were used for both soluble and cell-based immunoassays.

**Table 1:** Urogenital sample types used for detecting immune biomarkers

Sample type	Sex	*N*	Possible additives	Method	Application	Reference
CVL	F	24	Protease inhibitors	• 3–10 mL • Sterile saline or PBS • Repeatedly wash and aspirate fluid	Soluble & cell-based immune biomarkers • Antibodies (*n* = 8) • Cytokines (*n* = 17) • Cell phenotype (*n* = 3)	Ardizzone et al.^36^; Agrawal et al.^38^; Agrawal et al.^39^; Agrawal et al.^40^; Agrawal et al.^41^; Agrawal et al.^42^; Agrawal et al.^43^; Gupta et al.^44^; Jha et al.^45^; Mlisana et al.^46^; Ogendi et al.^47^; Spear et al.^48^; Sperling et al.^49^; Srivastava et al.^50^; Masson et al.^51^; Hwang et al.^52^; Richmond et al.^53^; Levine et al.^54^; Marconi et al.^55^; Jordan et al.^56^; Hedges et al.^57^; Barousse et al.^58^; Mott et al.^59^; Gupta et al.^60^
Cervical cytobrush	F	22	Antibiotics, fungicide, glutamine, FBS, sodium azide	• Collected in PBS, keratinocyte serum-free media, RPMI medium, or saline • Processed within 1–6 h • Cells can be dislodged using mechanical methods (vortex, rotate, or push through pipette point) or DTT treatment • Cell strainer (40–70 μm pore size) for homogenization^a^	Soluble & cell-based immune biomarkers • Antibodies (*n* = 1) • Cytokines (*n* = 15) • Cell phenotype (*n* = 16)	Albritton et al.^37^; Agrawal et al.^39^; Agrawal et al.^41^; Agrawal et al.^42^; Agrawal et al.^43^; Gupta et al.^44^; Jha et al.^45^; Srivastava et al.^50^; Masson et al.^51^; Levine et al.^54^; Gupta et al.^60^; Agrawal et al.^61^; Agrawal et al.^62^; Cohen et al.^63^; Ficarra et al.^64^; Ibana et al.^65^; Kelly et al.^66^; Scott et al.^67^; Vats et al.^68^; Reddy et al.^69^; Schust et al.^70^; McClure et al.^71^
Cervical swab	F	19	FBS (3%), 2X protease inhibitor cocktail, antibiotics, fungicide	• Cotton, nylon or Dacron-tipped swabs • Collected in PBS, Amplicor buffer (Roche), 2SP buffer or sterile saline—buffer not always mentioned • Left in place for 5–10 s while applying pressure or scraping^a^	Soluble immune biomarkers • Antibodies (*n* = 11) • Cytokines (*n* = 8)	Hedges et al.^57^; Reddy et al.^69^; Bua et al.^72^; Filardo et al.^73^; Fresse et al.^74^; Tsai et al.^75^; Fichorova et al.^76^; Fichorova et al.^77^; Witkin et al.^78^; Witkin et al.^79^; Zhang et al.^80^; Terho and Meurman^81^; Osser and Persson^82^; GrÖNroos et al.^83^; Honkonen et al.^84^; Kalimo et al.^85^; Puolakkainen et al.^86^; Witkin et al.^87^; Audu et al.^88^
Sponge	F	17	IGEPAL detergent (0.25%–10%), protease inhibitors, BSA (0.5%), Tween 20 (0.05%)	• Ophthalmic or nasal sponges • Elution method: immersing in medium or PBS, centrifugation using spin assembly apparatus, Spin-X microcentrifuge tube filters • Elution buffer: PBS, keratinocyte SFM^a^ • Some researchers block spin filters or equilibrate sponges using PBS with NaCl, aprotinin (a trypsin inhibitor), and sodium azide before centrifugation	Soluble immune biomarkers • Antibodies (*n* = 9) • Cytokines (*n* = 8)	Albritton et al.^37^; Sperling et al.^49^; Kelly et al.^66^; Darville et al.^89^; Wang et al.^90^; Wang et al.^91^; Ziklo et al.^92^; Ziklo et al.^93^; Poston et al.^94^; Darougar et al.^95^; Schachter et al.^96^; Thejls et al.^97^; Treharne et al.^98^; Omer et al.^99^; Lewis et al.^100^; Mardh et al.^101^; Southgate et al.^102^
Vaginal swab	F	5	IGEPAL detergent (10%), bacterial protease inhibitor (1%)	• Clinician collected or collected by self-sampling • Collected in sterile saline, PBS, or RNAlater (Ambion)—buffer not always mentioned	Soluble & cell-based immune biomarkers • Antibodies (*n* = 2) • Cytokines (*n* = 3) • Cell phenotype (*n* = 2)	Ziklo et al.^93^; Cauci and Culhane^103^; Chen et al.^104^; van den Broek et al.^105^; Cai et al.^106^
Urethrogenital swab	M/F	6	None	Calcium alginate swabs (Calgiswab)	Antibodies (*n* = 6)	Hammerschlag et al.^107^; McCormack et al.^108^; Ng et al.^109^; Gump et al.^110^; McCormack et al.^111^; McComb et al.^112^
Filter paper	F	4	None	Let paper absorb secretions and elute in PBS	Soluble immune biomarkers • Antibodies (*n* = 3) • Cytokines (*n* = 1)	Arno et al.^113^; Ruijs et al.^114^; Workowski et al.^115^; Brunham et al.^116^
Aspiration	F	5	None	Syringe, catheter, pipette, or Aspirette device	Soluble immune biomarkers • Antibodies (*n* = 4) • Cytokines (*n* = 1)	Cohen et al.^63^; Ruijs et al.^114^; Mahmoud et al.^117^; Persson et al.^118^; Mahmoud et al.^119^
Menstrual cup	F	2	None	• Self-sampling • SoftCup (Instead) • Specified timing^a^ • Diluted using PBS^a^	Soluble immune biomarkers • Antibodies (*n* = 1) • Cytokines (*n* = 1)	Abraham et al.^120^; Garrett et al.^121^
Cervical scraping	F	1	None	• Wooden spatula • Agitate spatula in saline	Cell phenotype (*n* = 1)	Levine et al.^54^
Total ejaculate	M	31	None	• By masturbation • Period of sexual abstinence (2–24 days)	Soluble & cell-based immune biomarkers • Antibodies (*n* = 12) • Cytokines (*n* = 7) • Cell phenotype (*n* = 13)	Bua et al.^72^; Cai et al.^122^; Martínez-Prado and Camejo Bermúdez^123^; Mazzoli et al.^124^; Dehghan Marvast et al.^125^; Karaulov et al.^126^; Pérez-Soto et al.^127^; Mazzoli et al.^128^; Hakimi et al.^129^; Hakimi et al.^130^; Habermann and Krause^131^; Moazenchi et al.^132^; Markelova et al.^133^; Gdoura et al.^134^; Samra et al.^135^; Penna Videau et al.^136^; El Feky et al.^137^; Ruijs et al.^138^; Dieterle et al.^139^; Eggert-Kruse et al.^140^; EzzEl-Din et al.^141^; Kojima et al.^142^; Munoz and Witkin^143^; Suominen et al.^144^; Weidner et al.^145^; Eggert-Kruse et al.^146^; Eggert-Kruse et al.^147^; Eggert-Kruse et al.^148^; Cai et al.^149^; Mazzoli et al.^150^; Bjercke and Purvis^151^
Seminal plasma	M	33	PureSperm (NidaCon Int.), PMSF protease inhibitor	• By centrifugation of total ejaculate • Centrifugation speed: 300–10,000 ×*g*^a^ • Centrifugation time: 5–10 min^a^	Soluble immune biomarkers • Antibodies (*n* = 27) • Cytokines (*n* = 11)	Martínez-Prado and Camejo Bermúdez^123^; Dehghan Marvast et al.^125^; Pérez-Soto et al.^127^; Habermann and Krause^131^; Moazenchi et al.^132^; Penna Videau et al.^136^; El Feky et al.^137^; Eggert-Kruse et al.^140^; EzzEl-Din et al.^141^; Munoz and Witkin^143^; Suominen et al.^144^; Weidner et al.^145^; Eggert-Kruse et al.^146^; Eggert-Kruse et al.^147^; Eggert-Kruse et al.^148^; Bjercke and Purvis^151^; Bollmann et al.^152^; Bollmann et al.^153^; Eggert-Kruse et al.^154^; Ochsendorf et al.^155^; Pérez-Soto et al.^156^; Kokab et al.^157^; Wolff et al.^158^; Wolff et al.^159^; Segnini et al.^160^; Motrich et al.^161^; Jungwirth et al.^162^; Nasr El-din et al.^163^; Munoz et al.^164^; Witkin et al.^165^; Yoshida et al.^166^; Gruschwitz et al.^167^
Urethral swab or smear	M	7	FBS (3%), antibiotics, fungicide	• Dacron-tipped stainless-steel shaft swabs, platinum wire loop swabs, cotton-tipped swabs, or calcium alginate urethrogenital swabs∗ • Swab medium: PBS, 2SP buffer^a^	Soluble & cell-based immune biomarkers • Antibodies (*n* = 5) • Cytokines (*n* = 1) • Cell phenotype (*n* = 2)	Fresse et al.^74^; Terho and Meurman^81^; Omer et al.^99^; Ng et al.^109^; Gdoura et al.^134^; Pate et al.^168^; Shahmanesh^169^
EPS	M	6	None	• By digital prostatic massage after voiding of urine • One author used a monofilament knitted polypropylene swab passed into the anterior urethra for collection of fluid after EPS expression	Antibodies (*n* = 6)	Cai et al.^122^; Mazzoli et al.^128^; Cai et al.^149^; Ostaszewska-Puchalska et al.^170^; Mardh et al.^171^; Shortliffe et al.^172^
FVU	M	3	Cytolyt, BSA (0.1%), DTT, Sputazol	• Initial flush of urine, diluted in PBS or PBS with additives^a^ • 5–40 mL • Early morning or collected after not having urinated for 3 hours^a^	Cell phenotype (*n* = 3)	Ito et al.^173^; Wiggins et al.^174^; Shahmanesh et al.^175^
PPM urine	M	3	None	• After rectal digital massage	Antibodies (*n* = 3)	Cai et al.^122^; Mazzoli et al.^128^; Cai et al.^149^
Other urine	M/F	6	None	• No further information	Soluble immune biomarkers • Cytokines (*n* = 2) • Antibodies (*n* = 2) • Cytokine mRNA (*n* = 2)	Bua et al.^72^; Tsai et al.^75^; Ochsendorf et al.^155^; Ohsawa et al.^176^; Ray et al.^177^; Ray et al.^178^

Sample type, sex, total number of studies (*N*) using a certain sample type, additives that can be added, collection method, application method used in provided references, and references (Refs.) are indicated in the table.

2SP, sucrose-phosphate buffer; F, female; FBS, fetal bovine serum; M, male; PPM urine, post-prostatic massage urine; SFM, serum-free media.

a Method not specified in all articles.

To collect male urogenital secretions, most studies used total ejaculate^72^^,^^122^^,^^123^^,^^124^^,^^125^^,^^126^^,^^127^^,^^128^^,^^129^^,^^130^^,^^131^^,^^132^^,^^133^^,^^134^^,^^135^^,^^136^^,^^137^^,^^138^^,^^139^^,^^140^^,^^141^^,^^142^^,^^143^^,^^144^^,^^145^^,^^146^^,^^147^^,^^148^^,^^149^^,^^150^^,^^151^ or seminal plasma.^123^^,^^125^^,^^127^^,^^131^^,^^132^^,^^136^^,^^137^^,^^140^^,^^141^^,^^143^^,^^144^^,^^145^^,^^146^^,^^147^^,^^148^^,^^151^^,^^152^^,^^153^^,^^154^^,^^155^^,^^156^^,^^157^^,^^158^^,^^159^^,^^160^^,^^161^^,^^162^^,^^163^^,^^164^^,^^165^^,^^166^^,^^167^ Less-common methods included urethral swab or smear,^74^^,^^81^^,^^99^^,^^109^^,^^134^^,^^168^^,^^169^ EPS,^122^^,^^128^^,^^149^^,^^170^^,^^171^^,^^172^ or urine samples,^72^^,^^75^^,^^122^^,^^128^^,^^149^^,^^155^^,^^173^^,^^174^^,^^175^ sometimes specified as FVU^173^^,^^174^^,^^175^ or post-prostatic massage urine.^122^^,^^128^^,^^149^ Total ejaculate was commonly used to assess both humoral and cellular immune responses, whereas cell-free seminal plasma was used exclusively to measure soluble immune mediators (antibodies and cytokines). EPS was used uniquely for measuring humoral immune responses. In men, urethral swabs and urine samples were used to evaluate both humoral and cellular immune responses.

### Detection of antibodies in urogenital secretions

**Figure 3:**
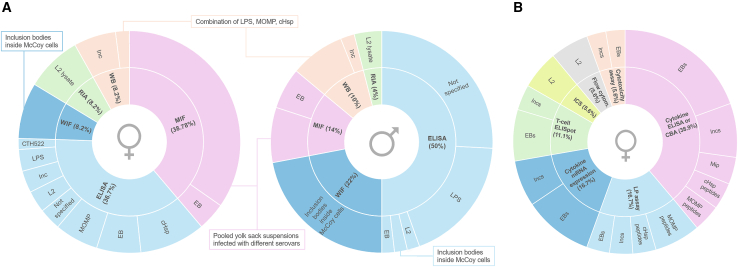
Immunoassays for detecting immune biomarkers in urogenital secretions Overview of different immunoassays for detecting immune biomarkers in urogenital secretions. Percentages indicate proportion of articles using the specified assay. Per immunoassay, different antigens that were used are given in the outer circle (proportional to the number of assays). CBA, cytokine bead array; Incs, inclusion proteins; LP assay, lymphoproliferation assay; L2, CT serovar L2 (lymphogranuloma venereum); Mip, macrophage infectivity potentiator; MOMP, major outer membrane protein; WB, western blot assay. (A) Overview of immunoassays used for detection of antibodies in female (left) and male (right) genital secretions. Studies including both female and male participants were included in both proportions. (B) Overview of different immunoassays used for measuring antigen-specific cellular immune responses in female urogenital secretions.

A variety of assays and antigens have been used for local antibody detection in urogenital secretions (Figure 3A). In men, 25 studies employed enzyme-linked immunosorbent assays (ELISA) to detect local CT-specific antibodies. Additionally, seven studies used microimmunofluorescence (MIF), 11 used whole-cell inclusion immunofluorescence (WIF), five used western blot, and two used radioimmunoassays (RIA). For antibody detection in female secretions, 18 studies used ELISA-based assays, 19 used MIF, four used WIF, four used western blot, and four used RIA. In total, 40 studies using immunofluorescence-based methods were identified in this review, including 24 using MIF and 16 using WIF.^181^^,^^182^^,^^183^^,^^184^ ELISA-based assays were used in 42 of the included studies, which are discussed in detail in Tables S3 and S4. One ELISA-based study included both female and male participants attending an STI clinic, but did not analyze them separately.^74^ Overall, western blot assays for detection of chlamydial antibodies were used in eight articles. Five of these studies used a commercial assay, the CT IgG + IgA western blot test AID (Autoimmun Diagnostika GmbH), including lipopolysaccharide (LPS), major outer membrane protein (MOMP), and chlamydial heat shock protein (cHsp) antigens.^106^^,^^122^^,^^124^^,^^128^^,^^149^ Three studies detected local antibodies against chlamydial inclusion membrane proteins (Incs) using an in-house immunoblot.^44^^,^^60^^,^^75^ RIAs were used in five articles for detection of CT-specific antibodies, all published in the 1980s. In these studies, L2 cell lysate coupled to polystyrene beads was used as antigen. Antibodies present in the sample were detected using radiolabeled anti-human antibodies.^81^^,^^83^^,^^84^^,^^85^^,^^144^

Functional antibody assays were performed in two studies, both focusing on female cervicovaginal secretions. The first study examined the inhibitory effects on inclusion formation using direct immunofluorescence-based staining of inclusion bodies. The study concluded that secretions of women with positive endocervical culture results exhibited higher anti-chlamydial activity compared with secretions of culture-negative women.^117^ More recently, a neutralization assay was performed by co-incubating serum- or CVL-derived antibodies with purified elementary bodies (EBs) on endocervical epithelial cell monolayers. Inclusion-forming units were then enumerated to assess neutralizing activity. The authors concluded that genital IgG, genital IgA, and serum IgA significantly inhibit the formation of inclusion bodies, whereas serum IgG did not. This finding may be explained by the higher affinity or broader reactivity of local mucosal antibodies. The study also investigated genogroup-specific neutralization and confirmed that the infecting strain can shape humoral immune responses.^36^

### Association between local and serum antibody levels

In this review, we identified eight studies that investigated associations between local and serum antibody levels. An overview of these studies is provided in Table 2. Strong correlations between cervical and serum IgG antibodies were observed in two studies (Spearman’s ρ = 0.89, Pearson’s R = 0.57), whereas correlations for IgA were notably weaker (Spearman’s ρ = 0.18, Pearson’s R = 0.26, respectively).^89^^,^^120^ Supporting these findings, agreement (κ-values) between IgG positivity in serum and vaginal swab samples was modest, and even lower for IgA in a fertility clinic cohort. However, this trend was not observed in an STI clinic cohort.^105^ In male urogenital secretions, only two studies found a significant association between serum and seminal plasma *Chlamydia* IgA positivity.^154^^,^^155^

**Table 2:** Relationship between local mucosal and serum antibody levels

Reference	Total *N*	Local secretion sample type	Isotype	Correlation between paired mucosal and serum samples	Significance level
Abraham et al.^120^	35	Menstrual cup	IgG	ρ = 0.89	*p* ≤ 0.0001
			IgA	ρ = 0.18	*p* = 0.43
Darville et al.^89^	151	Cervical sponge	IgG	R = 0.57	*p* = 1.82 E−13
			IgA	R = 0.26	*p* = 0.002
van den Broek et al.,^105^ cohort 1	77	Vaginal swab	IgG	κ = 0.130	*p* = 0.2
			IgA	κ = 0.37	*p* < 0.001
van den Broek et al.,^105^ cohort 2	116	Vaginal swab	IgG	κ = 0.27	*p* = 0.015
			IgA	κ = 0.17	*p* = 0.055
Eggert-Kruse et al.^154^	173	Seminal plasma	IgA	Chi-square or Fisher’s exact test	*p* < 0.001
Ochsendorf et al.^155^	125	Seminal plasma	IgA	Fisher’s exact test	*p* = 0.0003
			IgG	Fisher’s exact test	N.s.
Munoz et al.^164^	48	Seminal plasma, total ejaculate	IgA	Not reported	No correlation found
Shortliffe et al.^172^	82	Expressed prostatic secretions	IgA, IgG	Linear regression analysis	No correlation found
Weidner et al.^145^	131	Seminal plasma	IgA	Not reported	No correlation found

Correlation coefficients (Spearman correlation coefficient, ρ, or Pearson correlation coefficient, R) or agreement (κ value for positivity in serum and positivity in local antibodies) and significance level (*p* value) are given.

*N*, number of participants in the study.

### Detection of cellular immune responses in urogenital secretions

Overall, studies assessing antigen-specific immune responses in genitourinary secretions were limited (*n* = 9).^39^^,^^44^^,^^45^^,^^50^^,^^60^^,^^61^^,^^62^^,^^68^^,^^69^ An overview is provided in Table S5. Notably, one article focusing on cervical monocytes is included in this section.^62^ Although monocytes are not considered truly adaptive antigen-specific immune cells, they can play a major role in initiating both adaptive and innate immune responses against pathogens.^185^ Detection of cytokine-secreting cells following antigen stimulation by enzyme-linked immunosorbent spot (ELISpot) was performed in two articles.^44^^,^^61^ Another commonly used method for this endpoint is intracellular cytokine staining (ICS),^186^ which was applied in three studies; however, antigen-specific ICS was performed in only one of them.^45^^,^^51^^,^^69^ In total, 59 studies investigated cytokine and chemokine concentrations in urogenital secretions. Of these, 50 used ELISA-based techniques and 13 used cytokine magnetic bead assays. An overview of the number of studies measuring different cytokines and chemokines is presented in Figure S1. Seven studies measured cytokine concentrations following antigen stimulation using either cytokine ELISA or multiplex cytometric bead arrays.^44^^,^^45^^,^^50^^,^^60^^,^^61^^,^^62^^,^^68^ Three studies assessed lymphoproliferative responses following antigen stimulation. Two of these used radiolabeled ^3^H-thymidine incorporation into chromosomal DNA during mitosis for detection, whereas another employed a colorimetric MTT assay (3-(4,5-dimethyl thiazol-2-yl) 2, 5-diphenyl tetrazolium bromide) to determine stimulation indices.^39^^,^^44^^,^^68^ In this review, two studies were identified that involved cytotoxicity assays. The first study measured CT-specific cytotoxicity by quantifying lactate dehydrogenase release from damaged cells after antigen stimulation,^44^ whereas the second assessed non-antigen-specific cytotoxicity testing by measuring perforin expression of endocervical CD8^+^ T cells.^65^ Differential mRNA expression was reported in eight studies, three of which involved antigen stimulation.^44^^,^^60^^,^^61^^,^^62^^,^^67^^,^^93^^,^^177^^,^^178^ An overview of the antigens used for different antigen-specific T cell assays is presented in Figure 3B.

Seventeen studies used flow cytometry to determine immune cell phenotypes.^39^^,^^41^^,^^42^^,^^43^^,^^45^^,^^47^^,^^51^^,^^54^^,^^61^^,^^63^^,^^64^^,^^65^^,^^66^^,^^68^^,^^69^^,^^70^^,^^71^ Notably, this technique was exclusively applied to female urogenital secretions, with 16 studies analyzing cervical cytobrush samples and only one study using CVL.^47^ Only one study used flow cytometry to assess antigen-specific immune cell phenotypes.^69^ Overall, CD4^+^ T cells were analyzed in 14 studies, CD8^+^ lymphocytes in eight, and CD3 expression was explored in six studies. Some researchers also investigated additional surface markers.^47^^,^^54^^,^^64^^,^^65^^,^^66^ The CD4:CD8 T cell ratio was calculated in five articles. Dendritic cells (DCs) were analyzed in five articles. Some authors reported the percentage of lymphocytes among endocervical leukocytes (59%–86%, irrespective of infection or disease status),^39^^,^^41^^,^^71^ whereas others reported absolute cell counts. Multiple authors reported an increase in the mean number of CD4^+^ cells in CT-infected women, compared with those with fertility disorders or controls.^41^^,^^54^^,^^61^^,^^63^^,^^69^ This increase was further enhanced in women with mucopurulent cervicitis.^42^ Interestingly, only one study found a significant increase in the mean number of CD8^+^ mucosal T cells in CT-infected women compared with controls.^69^ B lymphocytes were present in low numbers, although they were only enumerated in two studies.^39^^,^^71^ Other methods for cell counting included microscopy-based techniques (*n* = 26) and the use of an automated urine particle analyzer (*n* = 1).^178^ In these studies, leukocytes or granulocytes were most commonly counted.

### Association between local and systemic cellular immune responses

Peripheral blood mononuclear cell (PBMC) samples were collected in 16 articles to evaluate systemic cellular immune responses. Some studies additionally collected whole blood, serum, or plasma to determine cytokine concentrations.

Flow cytometry data have demonstrated distinct differences between local and systemic cellular immune phenotypes. As expected, lymphocyte counts are lower in cytobrush samples compared with cervical biopsies and peripheral blood.^63^ Data on CD4^+^/CD8^+^ ratios in blood and endocervical samples are inconsistent, with some authors reporting comparable ratios and others showing a higher percentage of systemic CD4^+^ cells.^47^^,^^64^ Notably, the endocervix is predominantly populated by CD45RO^+^ effector memory T cells (TEM), as indicated by a significantly lower CD45RA^+^/CD45RO^+^ T cell ratio compared with peripheral blood.^64^^,^^65^^,^^66^ This TEM dominance was also observed in cervical CD8^+^ cells, which were notably less perforin positive than systemic TEM cells.^65^ Regarding CD4^+^ helper T cell populations, a distinct Th1/Th2 polarization has been reported. PBMCs show higher expression of Th2-associated chemokine receptor CCR4, whereas mucosal lymphocytes express more Th1-associated CXCR3 and CCR5. This suggests that CD4^+^ cells may undergo changes in polarization upon arrival at the site of infection. Additionally, mucosal homing and retention markers including CD103 (αE) are upregulated in cervical T cells compared with peripheral blood.^64^^,^^66^ Although some studies suggest that α4 and β7 co-expression is lower in cervical compared with systemic compartments, others report a unique population of cervical memory T cells co-expressing both mucosal and peripheral homing receptors (α4β7/CLA).^64^^,^^66^ During CT infection, endocervical T cells become highly activated and upregulate HLA-DR and CD38.^47^^,^^54^^,^^64^^,^^66^ Furthermore, the absolute number of tolerogenic plasmacytoid DCs per milliliter of blood was significantly lower in *Chlamydia-*infected women compared with their cervical samples and with blood samples from controls. This difference was even more pronounced in women with fertility disorders or mucopurulent cervicitis.^41^^,^^42^ The percentage of mature myeloid DCs (mDCs) in paired cervical and blood samples of CT-infected women was comparable; however, the absolute number of mDCs per milliliter of blood was significantly lower in CT-infected women without mucopurulent cervicitis compared with controls.^42^ Moreover, the percentage of granulocytes in endocervical samples was lower compared with paired PBMCs (49%–55% vs 61%–63%).^66^

When comparing lymphoproliferative responses of cervical lymphocytes and PBMCs after stimulation with cHsp and MOMP peptides, no significant differences between stimulation indices were observed,^39^ suggesting a good correlation between the two. Similar trends were observed for Inc-specific responses.^44^ In contrast, another study reported significantly higher proliferative responses of cervical lymphocytes compared with PBMCs following stimulation with MOMP peptides in CT-positive women.^68^ Several studies compared antigen-specific cytokine secretion by cervical lymphocytes and PBMCs. Jha et al. reported interferon (IFN)-γ secretion was comparable, whereas levels of interleukin (IL)-17 and IL-22 were slightly higher in cervical cell supernatants compared with PBMC supernatants from CT-infected patients.^45^ Upon MOMP stimulation, IL-6 and IL-10 levels were significantly lower in PBMC supernatants compared with supernatants from cervical lymphocytes in healthy control subjects. Conversely, in CT-positive women, IL-6 and IL-8 levels were significantly higher in the systemic compartment.^68^ When measuring IFN-γ concentrations directly in the local sample, strong correlations with plasma levels were lacking.^113^^,^^179^

Only two studies used data from both mucosal and systemic assays to calculate correlations. Neither study found significant correlations between cervicovaginal and systemic cytokine concentrations.^38^^,^^113^

### Factors influencing the detection of *Chlamydia trachomatis* immune biomarkers in urogenital secretions

**Figure 4:**
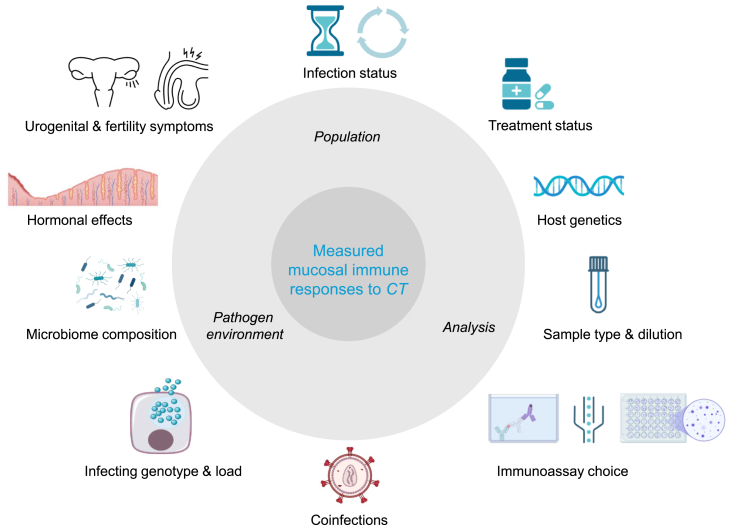
Different factors influencing mucosal immune responses to urogenital CT infections Overview of different factors affecting measured mucosal immune responses to urogenital *C. trachomatis* infections. Infection status (persistence vs. spontaneous clearance, primary exposure vs. past exposure, frequency of reinfections), treatment status (before or after treatment, treated or untreated), host genetics, sample type (explained in detail in Table 1) and dilution, immunoassay choice (antigen specific or not, species vs. genus specific, assay performance characteristics), presence of coinfections, infecting genotype and CT load, microbiome composition, hormonal effects (time point during menstrual cycle, menstruating vs. premenarche or postmenopausal, hormonal contraceptives), urogenital symptoms (symptomatic vs. asymptomatic, mucopurulent infection or not, etc.) and fertility symptoms (infertile vs. fertile) can all affect whether, which, and how many immune biomarkers are found in the urogenital secretions of men and women.

Besides sample type and immunoassay, we identified several host-pathogen factors that can influence the immune response measured during chlamydial infection. An overview of the major contributing factors is shown in Figure 4. Table 3 indicates the number of studies that accounted for different confounding factors, while details regarding their effects are presented in Table S6.

**Table 3:** Influencing factors taken into account in different papers

Influencing factor	N° of papers (% of total)	
Coinfections (tested or self-reported)	109 (75.2%^a^)	• *N* = 41 excluded coinfected participants, 68 did not. • 28/68 investigated effect of other infections on outcome parameters. • Effects on immune biomarkers in coinfected participants compared with monoinfection were investigated in 9 papers.
Microbiome	11 (7.6%^a^)	• 11 investigated effects of the microbiome; however, only 9 of them separately analyzed this for CT patients with and without microbiome parameters. • Two articles investigated effects of bacterial flora in men. • Seven articles investigated effects of bacterial vaginosis or female microbiome community state type.
Menstrual cycle	33 (37.1%^b^)	• One reported days since last menstruation • 14 did not collect samples during menstruation. • 18 specified timing: ◦ Midcycle, before ovulation: resembling late follicular phase (*n* = 11) ◦ Early follicular phase (*n* = 1) ◦ Pregnancy (*n* = 4) ◦ Oocyte retrieval for IVF (*n* = 2)
Other hormonal effects	5 (5.6%^a^)	• Two investigated effects of hormonal contraceptive use • Three investigated serum hormones (estradiol, progesterone)
Duration of infection (persistence, reinfections)	11 (7.6%^a^)	• Eight investigated effect on outcome parameters compared with primary infection or patients who cleared infection during follow-up
Clinical outcomes	34 (23.4%^a^)	• Fertility disorders (*n* = 22) • Urogenital tract symptoms including pain, discharge, inflammation (*n* = 12)

Number of papers out of total number of papers that accounted for coinfections, microbiome, menstrual cycle, hormonal effects, duration of infection, and clinical outcomes and details.

N° (%), number of papers (percentage); *N*, number, IVF, *in vitro* fertilization.

a All 145 total articles.

b 89 articles including women.

Although several studies investigated the independent effects of CT and other STIs on immune biomarkers, only nine explored the combined effects of coinfections compared with participants infected with CT alone. Interestingly, not all studies reported a significant impact of coinfections for each individual pathogen.^48^^,^^57^^,^^122^^,^^173^ Multiple studies included in this review indicate that CT coinfections with human papillomavirus (HPV), *Neisseria gonorrhea* (NG), and genital herpes (herpes simplex virus-2) can alter mucosal cytokine levels.^52^^,^^126^^,^^133^^,^^156^ Moreover, an increased risk of anti-*Chlamydia* antibody presence has been observed in men with a history of NG infection.^136^ Furthermore, four studies independently demonstrated that cervicovaginal cytokine patterns are distinctly associated with microbiome community state types or bacterial vaginosis (BV).^52^^,^^55^^,^^59^^,^^73^ In men, colonization with *Proteus* spp. has been associated with the presence of anti-*Chlamydia* IgA in seminal plasma.^154^ Nonetheless, not all authors found microbiome-associated differences in mucosal immune parameters.^56^^,^^64^^,^^103^^,^^172^

When collecting female urogenital samples, hormonal influences can be considered by measuring serum hormone levels or taking into account the menstrual cycle. Serum hormones may affect local immune cells present and cytokine concentrations measured.^38^^,^^41^^,^^177^ Additionally, local cytokine measurements can be influenced by hormonal contraceptive use.^77^

Multiple studies independently demonstrate that patients with recurrent or persistent infections, as well as those with fertility disorders, exhibit distinct differences in mucosal cytokine concentrations or mRNA expression.^38^^,^^39^^,^^40^^,^^41^^,^^43^^,^^56^^,^^90^^,^^93^^,^^94^^,^^104^ Discrepancies arise when comparing these findings with antigen-specific cytokine responses, which may vary depending on the antigen used for stimulation.^50^^,^^61^^,^^69^ Furthermore, lower cervical CD4^+^ T cell counts and differences in DC phenotypes have also been reported in women with fertility disorders.^41^^,^^43^^,^^61^ Interestingly, lymphoproliferative responses of cervical lymphocytes in women with recurrent infection appear to depend on the antigen used for stimulation.^39^ Regarding humoral responses, increased mucosal anti-*Chlamydia* antibody levels have been observed in women with recurrent infections or fertility disorders, although this varies depending on the antigen used and the cohort sampled.^39^^,^^40^^,^^43^^,^^50^^,^^78^^,^^79^^,^^82^^,^^83^^,^^105^^,^^144^^,^^154^ Although not the focus of this review, higher Toll-like receptor 2 expression on cervical monocytes has been observed in seropositive women, indicating previous CT infection, who already experienced fertility disorders.^62^ However, the overall number of cervical monocytes was lower in this group.^61^

Host genetics can also influence immune responses, as shown by Wang et al., who identified two *HLA* variants and a single *IL10* haplotype as independent markers of recurrent CT infection.^91^ Other key confounding factors include the infecting genotype and CT load. CT load can significantly impact immune cells present and local cytokine concentrations.^42^^,^^43^^,^^94^ Low levels of cervical (s)IgA and IgG have also been associated with high CT cervical load.^89^^,^^116^ It has long been known that antibody responses are serovar and genotype specific, but reinfection experiments have shown that antibody responses broaden over time until the infection is cleared. Surprisingly, secondary antibody responses are primarily directed against the serovar involved in the initial infection.^187^^,^^188^^,^^189^^,^^190^^,^^191^ One study included in this review found that local IgA is more broadly reactive against a range of CT *ompA* genotypes and may not only protect against the current infecting strain.^36^

## Discussion

Comprehensively, there has been growing scientific interest in investigating local mucosal immune responses rather than systemic responses.

**Figure 5:**
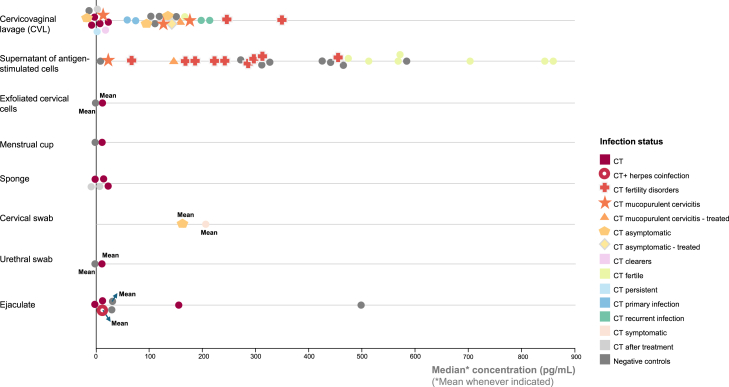
Measurement of urogenital interferon-γ concentration depends on infection status, immunoassay choice, and sample type Median IFN-γ concentrations (unless indicated as mean) in picograms per milliliter in different urogenital secretions retrieved from different studies (one data point indicates one population in a certain study). Infection status is indicated using the color and shape of data points. IFN-γ concentrations differ greatly when comparing sample types (cervicovaginal lavage, exfoliated cervical cells, menstrual cup, sponge, cervical swab, urethral swab, ejaculate) and CT infection status (CT [without further specifications], CT + *Herpes* coinfection, CT fertility disorders, CT mucopurulent cervicitis, CT mucopurulent cervicitis - treated, CT asymptomatic, CT asymptomatic - treated, CT clearers, CT fertile, CT persistent, CT primary infection, CT recurrent infection, CT symptomatic, CT after treatment, or negative uninfected controls) and depends on whether antigen-specific responses were considered (supernatant of antigen-stimulated cells) or not.

However, sampling the local environment presents several challenges, and differences between anatomical compartments and sample types should be considered in trial design. For example, proportional cell counts can differ between sample types.^54^^,^^192^^,^^193^ It should also be noted that superficial, apical immune cells might not be representative of cells residing in deeper layers of the tissue.^193^ In contrast, more invasive scraping or brushing techniques may collect circulating blood-derived cells rather than mucosal cells. The impact of blood contamination remains unclear, as even in visually contaminated samples most cells exhibit a mucosal phenotype. Lymphocytes in cervical samples are often outnumbered by other cell types including epithelial cells and neutrophils, which can hinder detection.^192^^,^^193^ Notably, although cells sampled through cervical cytobrushes are referred to as “cervical cells,” their exact anatomical origin is difficult to determine.^192^ In the context of cytokine quantification, variations in sample types can lead to inconsistent results. For instance, significantly higher tumor necrosis factor-α concentrations were observed in vaginal sponge samples before treatment compared with after treatment, whereas CVL samples showed no variations upon treatment.^49^ Discrepancies in cytokine concentrations are even more pronounced when comparing cytokine concentrations in the raw urogenital secretions with those in antigen-stimulated cervical cell supernatants (Figure 5). Sample-dependent differences have also been described for antibody detection.^21^^,^^37^^,^^194^ Table 4 provides an overview of different sample types and their suitability in different scenarios. This can help guide the selection of appropriate sample types depending on the study population, the immunological biomarker of interest, and whether self-collection or integrated DNA detection is desired. Combining DNA detection with both cellular and humoral immune biomarkers from a single sample offers complementary insights into mucosal immunity, enabling a more comprehensive understanding of local immune responses. In sample types that allow antibody detection, antibodies can be used to assess past exposure (or vaccination).^214^^,^^215^^,^^216^^,^^217^^,^^218^^,^^219^^,^^220^ Detecting pathogen-specific DNA alongside corresponding immunological endpoints is feasible in various sample types, including vaginal and cervical swabs, cervical cytobrushes, urethral or urethrogenital swabs, ejaculate, and FVU. Among these, vaginal swabs, ejaculate and (first-void) urine can be self-collected. Interestingly, FVU is a promising self-collectable sample type for evaluating HPV-related (functional) immune biomarkers and DNA in women.^19^^,^^204^^,^^221^

**Table 4:** Adequacy of distinct urogenital sample types for measuring different mucosal biomarkers

Sample type	Sex	Self-collection	NAAT	Antibody detection	Functional antibodies	Cytokines	Cellular	Remarks	Reference
CVL	F	+	–	+++	++	+++	+	• Recommended sample for analysis of soluble immune factors. • Suitable for both soluble and cell-based assays. • Self-sampling devices available (e.g., Delphi Screener, Pantarhei Screener); however, not commonly used.	World Health Organization^28^; Ardizzone et al.^36^; Agrawal et al.^38^; Agrawal et al.^39^; Agrawal et al.^40^; Agrawal et al.^41^; Agrawal et al.^42^; Agrawal et al.^43^; Gupta et al.^44^; Jha et al.^45^; Mlisana et al.^46^; Ogendi et al.^47^; Spear et al.^48^; Sperling et al.^49^; Srivastava et al.^50^; Masson et al.^51^; Hwang et al.^52^; Richmond et al.^53^; Levine et al.^54^; Marconi et al.^55^; Jordan et al.^56^; Hedges et al.^57^; Barousse et al.^58^; Mott et al.^59^; Gupta et al.^60^; de Melo Kuil et al.^195^; Verhoef et al.^196^
Cervical cytobrush	F	+^a^	++	+	?	++	+++	• Care should be taken to avoid bleeding. • Recommended sample for analysis of cells.	World Health Organization^28^; Albritton et al.^37^; Agrawal et al.^39^; Agrawal et al.^41^; Agrawal et al.^42^; Agrawal et al.^43^; Gupta et al.^44^; Jha et al.^45^; Srivastava et al.^50^; Masson et al.^51^; Levine et al.^54^; Gupta et al.^60^; Agrawal et al.^61^; Agrawal et al.^62^; Cohen et al.^63^; Ficarra et al.^64^; Ibana et al.^65^; Kelly et al.^66^; Scott et al.^67^; Vats et al.^68^; Reddy et al.^69^; Schust et al.^70^; McClure et al.^71^; McKinnon et al.^192^; Lund et al.^193^; Leinonen et al.^197^
Cervical swab	F	+^a^	++	++	+	++	?	• Functional (neutralizing) antibodies demonstrated in other infections.	World Health Organization^28^; Hedges et al.^57^; Reddy et al.^69^; Bua et al.^72^; Filardo et al.^73^; Fresse et al.^74^; Tsai et al.^75^; Fichorova et al.^76^; Fichorova et al.^77^; Witkin et al.^78^; Witkin et al.^79^; Zhang et al.^80^; Terho and Meurman^81^; Osser and Persson^82^; GrÖNroos et al.^83^; Honkonen et al.^84^; Kalimo et al.^85^; Puolakkainen et al.^86^; Witkin et al.^87^; Audu et al.^88^; Leinonen et al.^197^; Draper et al.^198^
Sponge	F	–	–	++	+	++	?	• Functional (neutralizing) antibodies demonstrated in other infections.	Kemp et al.^21^; World Health Organization^28^; Albritton et al.^37^; Sperling et al.^49^; Kelly et al.^66^; Darville et al.^89^; Wang et al.^90^; Wang et al.^91^; Ziklo et al.^92^; Ziklo et al.^93^; Poston et al.^94^; Darougar et al.^95^; Schachter et al.^96^; Thejls et al.^97^; Treharne et al.^98^; Omer et al.^99^; Lewis et al.^100^; Mardh et al.^101^; Southgate et al.^102^
Vaginal swab	F	++	+++	+	?	+	+	• Recommended sample for NAAT testing in women. • Can be self-collected or clinician collected.	World Health Organization^28^; Ziklo et al.^93^; Cauci and Culhane^103^; Chen et al.^104^; van den Broek et al.^105^; Cai et al.^106^
Filter paper	F	–	–	+	?	+	?		World Health Organization^28^; Arno et al.^113^; Ruijs et al.^114^; Workowski et al.^115^; Brunham et al.^116^
Aspiration	F	–	–	+	?	+	?		World Health Organization^28^; Cohen et al.^63^; Ruijs et al.^114^; Mahmoud et al.^117^; Persson et al.^118^; Mahmoud et al.^119^
Menstrual cup	F	++	–	++	+	++	+	• Recently shown that it can be used for isolation of FGT immune cells. • Functional (neutralizing) antibodies. demonstrated in other infections.	World Health Organization^28^; Abraham et al.^120^; Garrett et al.^121^; Peters et al.^199^; Mkhize et al.^200^
Cervical scraping	F	–	–	?	?	?	+	• Care should be taken to avoid bleeding.	World Health Organization^28^; Levine et al.^54^; McKinnon et al.^192^; Lund et al.^193^
Total ejaculate	M	++	++	++	+	+	++	• Total ejaculate is expected to contain the same functional antibodies when compared with seminal plasma.	World Health Organization^28^; Bua et al.^72^; Cai et al.^122^; Martínez-Prado and Camejo Bermúdez^123^; Mazzoli et al.^124^; Dehghan Marvast et al.^125^; Karaulov et al.^126^; Pérez-Soto et al.^127^; Mazzoli et al.^128^; Hakimi et al.^129^; Hakimi et al.^130^; Habermann and Krause^131^; Moazenchi et al.^132^; Markelova et al.^133^; Gdoura et al.^134^; Samra et al.^135^; Penna Videau et al.^136^; El Feky et al.^137^; Ruijs et al.^138^; Dieterle et al.^139^; Eggert-Kruse et al.^140^; EzzEl-Din et al.^141^; Kojima et al.^142^; Munoz and Witkin^143^; Suominen et al.^144^; Weidner et al.^145^; Eggert-Kruse et al.^146^; Eggert-Kruse et al.^147^; Eggert-Kruse et al.^148^; Cai et al.^149^; Mazzoli et al.^150^; Bjercke and Purvis^151^
Seminal plasma	M	–	–	+	+	+	–	• Centrifugation step necessary, making it less practical. • Inherently does not contain any cells. • Functional (ADCC-enhancing) antibodies demonstrated in other infections.	World Health Organization^28^; Martínez-Prado and Camejo Bermúdez^123^; Dehghan Marvast et al.^125^; Pérez-Soto et al.^127^; Habermann and Krause^131^; Moazenchi et al.^132^; Penna Videau et al.^136^; El Feky et al.^137^; Eggert-Kruse et al.^140^; EzzEl-Din et al.^141^; Munoz and Witkin^143^; Suominen et al.^144^; Eggert-Kruse et al.^145^; Eggert-Kruse et al.^146^; Eggert-Kruse et al.^147^; Eggert-Kruse et al.^148^; Bjercke and Purvis^151^; Bollmann et al.^152^; Bollmann et al.^153^; Eggert-Kruse et al.^154^; Ochsendorf et al.^155^; Pérez-Soto et al.^156^; Kokab et al.^157^; Wolff et al.^158^; Wolff et al.^159^; Segnini et al.^160^; Motrich et al.^161^; Jungwirth et al.^162^; Nasr El-din et al.^163^; Munoz et al.^164^; Witkin et al.^165^; Yoshida et al.^166^; Gruschwitz et al.^167^; Jecht and Poon^201^; Parsons et al.^202^
Urethral swab/smear	M	–	++	+	?	+	+		World Health Organization^28^; Fresse et al.^74^; Terho and Meurman^81^; Omer et al.^99^; Ng et al.^109^; Gdoura et al.^134^; Pate et al.^168^; Shahmanesh^169^
EPS	M	–	–	++	?	+	+	• It has been demonstrated that immune cells and cytokines can be measured in EPS in other contexts (e.g., prostatitis).	World Health Organization^28^; Cai et al.^122^; Mazzoli et al.^128^; Cai et al.^149^; Ostaszewska-Puchalska et al.^170^; Mardh et al.^171^; Shortliffe et al.^172^; Nadler et al.^203^
FVU	M/F	++	+++	++	++	++	++	• In men: recommended sample for NAAT and for analysis of soluble and cell-based immune mediators (more practical compared with ejaculate). • In women: up to 10% less sensitive for NAAT compared with vaginal or endocervical swabs. In other genital infections, non-inferiority for NAAT and presence of functional (neutralizing) antibodies has been shown.	World Health Organization^28^; Ito et al.^173^; Wiggins et al.^174^; Shahmanesh et al.^175^; Teblick et al.^204^; Centers for Disease Control and Prevention^205^; Aaron et al.^206^; Van Keer et al.^207^
PPM urine	M	–	–	++	?	+	+	• It has been demonstrated that immune cells, cytokines, and antibodies can be measured in PPM urine in other contexts (e.g., UTI, prostatitis)	World Health Organization^28^; Cai et al.^122^; Mazzoli et al.^128^; Cai et al.^149^; Ludwig et al.^208^; Fujita et al.^209^; Cao et al.^210^
Midstream urine	M/F	++	++	+	+	++	+	• It has been demonstrated that immune cells, cytokines and antibodies can be measured in midstream urine in other contexts (e.g., UTI).	World Health Organization^28^; Ludwig et al.^208^; Kellogg et al.^211^; Gibb and Edmond^212^; Drage et al.^213^
Urethrogenital swab	M/F	–	++	+	?	?	?		World Health Organization^28^; Hammerschlag et al.^107^; McCormack et al.^108^; Ng et al.^109^; Gump et al.^110^; McCormack et al.^111^; McComb et al.^112^

Sample type; sex it can be used for; self-collection potential; whether NAAT is available^28^; if antibody detection, cytokine measurement, and isolation of cells for cell-based assays is possible; and remarks are indicated.

+/++/+++, can be used for this endpoint; -, not suitable for this endpoint; ?, no indications whether this sample is suitable for the endpoint or not.

F, female; FGT, female genital tract; M, male; NAAT, nuclear acid amplification test; PPM urine, post-prostatic massage urine; UTI, urinary tract infection.

a Cervicovaginal swabs and brushes can be self-collected, whereas endocervical swabs and brushes are always clinician collected.

Despite sampling challenges, researchers have demonstrated that CD69 and CD103 expressions are significantly higher in female reproductive tract tissues compared with blood, indicating the presence of cells with a TRM phenotype, even in a healthy population.^193^ After CT infection, noncanonical CD4γ13 TRM cells producing both IFN-γ and IL-13 are thought to expand. These memory T cells remain within lymphoid aggregates or memory lymphoid clusters in the genital tract after pathogen clearance by upregulating CD69 expression, alongside B cells and macrophages, which act as antigen-presenting cells. TRM cells are essential for a swift response to CT reinfection through local expansion, and provide superior protection compared with systemic CD4^+^ T cells.^32^^,^^33^^,^^34^^,^^35^ Although IFN-γ responses are commonly considered when selecting cytokine biomarkers, IL-13-related responses appear under-represented in the scientific literature, with only one study reporting significant findings.^126^ In men, CD103^+^ CD8^+^ TRM cells have been identified in the penile urethra and TRM cells have also been observed in the interstitial space of the testis, although evidence is sparse.^222^ No studies included in this review investigated CD69-expressing cells in genitourinary secretions. Most, instead, focused on cytokine concentrations. Interpreting these findings is challenging, as studies used different sample types and often compared heterogeneous populations in terms of infection status (e.g., duration of infection, primary infection vs. recurrent infection, before vs. after treatment, coinfections or not) (Figure 5). Moreover, most cytokines exhibit a protective optimum concentration range that facilitates pathogen clearance, whereas excessive levels can trigger inflammatory reactions and immunopathological tissue damage. Cytokine effects therefore depend on their concentration, immune microenvironment, and the stage of infection.^223^^,^^224^^,^^225^^,^^226^ Several studies also explored mucosal immune cells, most in a non-antigen-specific manner.

Nine studies investigating antigen-specific cellular responses were included in this review. Despite ELISpot being considered a highly sensitive, standardizable method for use with PBMCs, only two of the nine studies employed this technique.^186^ Given its adaptability to various (vaccine) antigens, ELISpot holds promise for measuring local immune responses in CT vaccine trials. T cell ELISpot has already been applied in human respiratory,^227^^,^^228^ gastrointestinal,^229^^,^^230^^,^^231^ and even cervicovaginal tissues,^15^^,^^232^ highlighting its suitability for mucosal applications. Notably, the two trials in this review that used ELISpot both relied on cervical cytobrush samples and excluded specimens with insufficient cervical cells or lymphocytes (fewer than 1–2 million cells/mL), suggesting that cell yield is a limiting factor.^44^^,^^61^ The low purity and yield, as well as contamination with either blood-derived cells or foreign pathogens and particles, have also been reported in other mucosal contexts, such as nasal T cells.^233^ Other techniques used to assess antigen-specific cellular immune responses included cytokine ELISA and mRNA expression in the supernatant of cultured cervical lymphocytes, lymphoproliferation assays, and cytotoxicity assays.^39^^,^^44^^,^^45^^,^^50^^,^^60^^,^^61^^,^^62^^,^^68^ Only one study employed flow cytometry or ICS.^69^ State-of-the-art techniques such as peptide-major histocompatibility complex multimers and T cell receptor sequencing have not yet been applied to assess antigen-specific cellular immune responses in the context of CT, although they have been performed in other diseases.^234^^,^^235^^,^^236^^,^^237^^,^^238^ Despite strong evidence that circulating T cells, particularly IFN-γ-producing CD4^+^ Th1 cells, play a key role in controlling CT infection,^239^^,^^240^^,^^241^^,^^242^^,^^243^^,^^244^^,^^245^^,^^246^^,^^247^^,^^248^ only two studies investigated correlations between local and systemic CT-specific cellular immune responses and neither reported significant associations.^38^^,^^113^

Several assays are available for detecting anti-CT serum antibodies.^182^ In this review, we investigated the use of these assays for detecting local antibodies in urogenital secretions. Multiple earlier studies used immunofluorescence-based assays, which were once considered the gold standard.^182^ However, these assays exhibit cross-reactivity with other *Chlamydia* spp., have low sensitivity, and require subjective microscopic interpretation, reducing diagnostic reproducibility.^182^ The RIA technique has largely been abandoned too due to cost, safety, and waste issues.^249^ More sensitive, specific, and safer ELISA-based techniques are now preferred. Notably, no studies included in this review employed more recent Luminex bead-based or luciferase immunosorbent assays.^182^ To quantify local antibody responses, various antigens were used, including whole EBs, recombinant cHsps, recombinant Incs, LPS, and MOMP proteins. However, none of these antigens are fully species specific to CT, which compromises specificity due to cross-reactivity.^250^^,^^251^^,^^252^^,^^253^ For cHsp in particular, cross-reactivity with human heat shock proteins has been suggested due to sequence homology.^254^^,^^255^ Despite this, immunoassays using whole EBs have demonstrated good sensitivity compared with MOMP-based ELISAs.^256^^,^^257^ One study used CTH522, a recombinant version of CT MOMP, to measure vaccine-induced antibodies.^120^^,^^258^^,^^259^ However, not all authors report the antigen used. For example, Ezz-El-Din and coworkers used a combination of antigens including MOMP, and two immunodominant species-specific antigens chlamydial protease-like activity factor and translocated actin-recruiting phosphoprotein, although this was not explicitly stated in the article.^141^^,^^260^^,^^261^ Notwithstanding its status as a superior antigen, as evidenced by its high immunogenicity and its lack of crossreaction with *Chlamydia pneumoniae*,^182^^,^^262^ no articles describing plasmid glycoprotein 3-based assays in urogenital secretions were retrieved in the present review.

The role of antibody-mediated immune responses in human *Chlamydia* infection is paradoxical. It has serum anti-CT IgG, and cervical anti-CT IgA and IgG have been shown to correlate with reduced cervical burden. However, the presence of both cervical and serum IgG has also been associated with an increased risk of incident infection. Antibodies alone are insufficient to prevent ascension.^89^ High antibody titers are often linked with enhanced disease severity, reflecting repeated or prolonged exposure rather than protective immunity.^263^^,^^264^ Proposed protective mechanisms include neutralization, antibody-dependent cellular cytotoxicity (ADCC), and antibody-mediated phagocytosis.^262^^,^^265^^,^^266^^,^^267^^,^^268^ In neutrophils, an additional role for complement receptor-mediated phagocytosis rather than FcγR receptor-mediated phagocytosis has also been suggested.^266^ Unfortunately, this literature search did not identify any studies investigating mucosal phagocytosis or ADCC enhancement by local antibodies. However, Ardizzone et al. demonstrated that mucosal antibodies in female genital secretions more efficiently neutralize EBs compared with serum-derived antibodies at equivalent concentrations, highlighting important functional differences between local and systemic antibody responses.^36^ Further research is warranted to elucidate the functional role of mucosal antibodies compared with serum antibodies. Only two studies investigated correlations between local and serum antibodies. Strong correlations for IgG support the notion that cervical IgG is a composite of both transudated serum IgG and locally produced IgG, whereas weaker correlations for IgA suggest that mucosal IgA is primarily produced locally by plasma cells.^89^^,^^120^ One study reported a difference in κ-agreement between an STI clinic vs. fertility clinic cohort and suggests that mucosal IgA is associated with tubal pathology in the latter.^105^ More research is needed to clarify the functionality of local antibodies and their correlation with serum antibodies in male genital secretions.

Several factors are known to influence immune responses to CT infection. One of the most critical is the microbiome. Urogenital CT strains can utilize a specific enzyme to convert indole (produced by, e.g., *Prevotella* spp., which are elevated in BV) back into tryptophan. This essential amino acid would otherwise be depleted under the influence of IFN-γ.^225^^,^^269^ However, the role of the microbiome in male CT infections remains poorly understood.^270^^,^^271^^,^^272^ Another major confounding factor is the presence of coinfections.^264^^,^^273^^,^^274^ Studies that did not exclude coinfected participants observed several pathogen-specific differences in mucosal cytokine responses, with varying combinations of coinfections producing distinct cytokine profiles. This underscores the importance of combined STI screening and the use of antigen-specific immunoassays. Additional confounding variables are host genetics,^274^^,^^275^^,^^276^^,^^277^^,^^278^ hormonal influences,^279^^,^^280^ the infecting genotype, and CT bacterial load.^36^^,^^42^^,^^43^^,^^89^^,^^94^^,^^116^^,^^187^^,^^188^^,^^189^^,^^190^ The characteristics of the study population, such as treatment status, clinical presentation, and fertility status, also significantly affect study outcomes. Persistent or recurrent infections can provoke an aberrant inflammatory response inducing fibrosis and tissue scarring, leading to reproductive sequelae. These pathological outcomes may also skew immune responses measured.^32^^,^^274^^,^^281^^,^^282^^,^^283^^,^^284^ Therefore, all these factors should be carefully considered during participant recruitment and when interpreting trial results.

### Future directions

As urogenital sampling for detection of mucosal immune biomarkers has gained increasing interest, we propose several aspects that should be considered and optimized for future research in this field. First, we suggest that sample collection and processing be standardized across research groups to enable meaningful comparisons. Therefore, it is important to disclose detailed information regarding sample collection, processing, and dilution. Furthermore, protocols for isolation of cervical mononuclear cells should be harmonized, whereas protocols for isolation of male urogenital mononuclear cells still need to be developed. Uniform collection methods for measurement of local antibodies should also be agreed upon, with normalization for differences in sample volumes and dilution factors. Second, we believe that consensus regarding “gold-standard” assays and antigens for both humoral and cellular immune responses is essential. For validating assays that measure humoral immune responses, a reference standard using either antigen-specific monoclonal antibodies or a serum standard should be available.^214^ When measuring cellular immune responses, the focus should be on antigen-specific responses, as it seems impossible to account for all confounding factors affecting the local immune environment. Therefore, a reference antigen and a shared protocol for stimulating immune cells in antigen-specific testing would be highly beneficial. Third, differences between anatomical compartments and systemic immune responses should be further investigated and correlations should be evaluated. This can only be achieved when immunoassays are validated for the sample type used, ensuring equivalent assay performance between local and systemic compartments. This, combined with basic experimental research, will enhance our physiological understanding of how antibodies and immune cells reach the primary site of infection. Finally, long-term follow-up of both mucosal and systemic immunological endpoints, along with additional clinical endpoints, is needed to define a correlate of protection, which will be of paramount importance evaluating future vaccine efficacy.

### Limitations of the study

Despite these interesting findings, it is important to acknowledge potential limitations in our study. This review did not consider clinical endpoints associated with proposed immunological endpoints, even though they are essential for defining a correlate of protection. However, we did highlight the effect of fertility disorders and urogenital symptoms as factors that can influence mucosal CT immunity. We did not investigate the effect of other confounding factors including demographic background or immune-related comorbidities. The data collected are highly variable regarding population characteristics, sample type and processing, specific biomarkers assessed, and detection methods. Therefore, a meta-analysis was unfortunately not feasible. Yet, the data are presented in a systematic way, and an overview is provided to allow comparison between the studies.

## Resource availability

### Lead contact

Requests for further information and resources should be directed to and will be fulfilled by the lead contact, Anne Van Caesbroeck (anne.vancaesbroeck@uantwerpen.be).

### Materials availability

This study did not generate new unique reagents.

### Data and code availability

• Data reported in this paper will be shared by the lead contact upon request.
• This paper does not report original code.
• Any additional information required to reanalyze the data reported in this paper is available from the lead contact upon request.

## Acknowledgments

Our sincere gratitude goes to all authors willing to provide additional information or raw data related to studies included in this review. This work was supported by 10.13039/501100007660Universiteit Antwerpen (TT(ZAP)BOF 542300005) and the 10.13039/501100000780European Union (ERC, URISAMP, 101040588). E.v.d.B. is supported by a PhD fellowship of the Research Foundation Flanders (Belgium) (10.13039/501100003130FWO; grant number 1SC9125N). Views and opinions expressed are, however, those of the authors only and do not necessarily reflect those of the European Union or the European Research Council Executive Agency. Neither the European Union nor the granting authority can be held responsible for them.

## Author contributions

Conceptualization, A.V. and L.T.; methodology, A.V.C., A.V., L.T., M.L., and E.v.d.B.; investigation, A.V.C., M.L., and E.v.d.B.; writing—original draft, A.V.C.; writing—review & editing, A.V.C., A.V., L.T., E.v.d.B., M.L., and P.P.; funding acquisition, A.V.; resources, A.V.; supervision, A.V., L.T., and P.P.

## Declaration of interests

The authors declare no competing interests.

## Declaration of generative AI and AI-assisted technologies in the writing process

During the preparation of this work, the authors used systematic review management platform Ryyan^285^ to screen titles and abstracts retrieved in the database search based on inclusion and exclusion criteria. While using this tool, the authors reviewed and edited the content as needed and take full responsibility for the content of the publication.

## STAR★Methods

### Key resources table

**Table:** 

REAGENT or RESOURCE	SOURCE	IDENTIFIER
**Software and algorithms**		

Ryyan	Ouzzani et al., 2016	https://doi.org/10.1186/s13643-016-0384-4
Microsoft Excel	Microsoft	RRID:SCR_016137
EndNote	Clarivate Analytics	RRID:SCR_014001
Biorender	BioRender	RRID:SCR_018361

### Experimental model and study participant details

A literature search was conducted through Ovid MEDLINE, Web of Science, and Scopus databases on October 9^th^ 2023, and updated on February 11^th^ 2025. The main search concepts were urogenital secretions in men and women, *Chlamydia trachomatis*, and humoral and cellular immune responses. Animal studies were excluded using predefined filters for each database^286^^,^^287^^,^^288^ and studies were limited to English language. No limits regarding publication period were applied. The detailed search strategy is provided in Table S1. Duplicate records were removed using EndNote. Additional articles were manually searched through screening of reference lists of the articles identified via database search.

### Method details

#### Selection criteria

Articles reporting on the detection of CT-specific antibodies or cellular immune responses in urogenital secretions were included. Further inclusion criteria were as follows: (1) English language, (2) Clinical trials, cohort studies, case-control studies, cross-sectional studies, letters to the editor, case reports and case series. Non-human studies, conference proceedings, reviews and meta-analyses were excluded. Articles were excluded if data were incomplete, and the author could not be reached to provide additional information regarding study protocol or outcome. Additionally, studies solely reporting on innate immune biomarkers were excluded. Studies reporting on cytokine concentrations were considered for inclusion, as changing cytokine environments can be mediated by both the innate and the adaptive immune system. Articles reporting on lymphocytes and CD45^+^ leukocytes (as the latter can include both innate and adaptive immune cells) were included as well. In addition, studies reporting on STIs overall or *Chlamydia*-associated diseases, without separate analysis of CT-infected or coinfected participants were excluded, unless the author was successfully contacted and could provide additional information.

A prospective protocol was registered on PROSPERO (CRD42023470875) on October 27^th^ 2023, following PRISMA guidelines.^289^ Every record was screened independently by at least two authors and in case of disagreement, consensus was reached internally or by consulting a third reviewer. Risk of bias was assessed for every selected article. Appropriate JBI critical appraisal tools were used for cross-sectional studies, case reports, case series, randomized controlled trials and letters to the editor.^290^^,^^291^^,^^292^^,^^293^ For case-control and cohort studies, the Newcastle-Ottawa Scale was used.^294^

#### Data extraction, synthesis and presentation of the results

From each eligible article, data related to: (a) population characteristics, (b) CT antigen detection method used, (c) coinfections assessed, (d) sample type and collection method, (e) local immune parameters measured, and their quantification (f) laboratory techniques used for assessment of immune parameters, (g) systemic immune parameters measured and their correlation to local parameters, if available, (h) clinical signs and symptoms, and (i) relevant conclusions of the study were collected.

### Quantification and statistical analysis

This study did not involve any statistical analyses.

### Additional resources

PROSPERO protocol: https://www.crd.york.ac.uk/PROSPERO/view/CRD42023470875.

## Supplemental information

Document S1. Figure S1 and Tables S1, S2, and S6

Table S3. Overview of studies on detection of antibodies in male urogenital secretions using ELISA-based assays

Table S4. Overview of studies on detection of antibodies in female urogenital secretions using ELISA-based assays

Table S5. Overview of studies on detection of antigen-specific adaptive cellular immune responses in urogenital secretions
