# Supplementary material for: Unveiling Chlamydia trachomatis immunity in urogenital secretions: A systematic review
Source: iScience. 2025 Aug 28;28(10):113453. doi: 10.1016/j.isci.2025.113453 (PMC12478110; doi:10.1016/j.isci.2025.113453)
Supplement: Document S1. Figure S1 and Tables S1, S2, and S6 [file mmc1.pdf]

iScience, Volume *28*

Supplemental information

Unveiling *Chlamydia trachomatis* immunity in urogenital secretions: A systematic review

Anne Van Caesbroeck, Marijana Lipovac, Eef van den Borst, Paolo Palma, Laura Téblick, and Alex Vorsters

SUPPLEMENTAL INFORMATION

Table S1: Search strategy for different databases used.

**MEDLINE (Ovid)**	
Search	Query
1.	(meatal* or penile* or semen or smegma or urethral*).mp.
2.	(cervicovaginal secretion* or vagina* or cervi* or muc*).mp.
3.	Urethritis/ or Urinary Tract Infections/ or Penis/ or Urethra/ or Cervix Mucus/ or Semen/ or Smegma/ or Urine/ or Vagina/ or Vulva/ or Vaginal Discharge/
4.	(first* urine or urine or mucus or urogenital discharge or urogenital secretion* or genital discharge or genital secretion*).mp.
5.	1 or 2 or 3 or 4
6.	(Chlamydia* or trachomatis).mp.
7.	Chlamydia/ or *Chlamydia Infections/ or *Chlamydia trachomatis/
8.	6 or 7
9.	(antibod* or immunoglobulin* or Ig*).mp.
10.	(T-cell* or B-cell* or CD* or helper*cell or Th22* or cytokine* or IL* or interleukin* or interferon* or Treg* or Trm*).mp.
11.	exp Adaptive Immunity/ or exp Immunity, Innate/ or Immunity, Mucosal/ or exp Immune System/ or Immunoglobulins/ or Antibodies/ or Immunoglobulin A/ or Immunoglobulin G/ or Immunoglobulin M/ or B-Lymphocytes/ or T-Lymphocytes/ or CD4-Positive T-Lymphocytes/ or CD8-Positive T-Lymphocytes/ or Cytokines/ or Th1 Cells/ or Th2 Cells/ or Th17 Cells/ or Interferons/
12.	9 or 10 or 11
13.	5 and 8 and 12
14.	exp animals/ not humans.sh.
15.	13 not 14
16.	limit 15 to English language
**Web of Science (Clarivate)**	
Search	Query
1.	ALL=("meatal*" or "penile*" or "semen" or "smegma" or "urethra*" or "cervicovaginal secretion*" or "vagina*" or "cervi*" or "muc*" or "urethritis" or "urinary tract infection*" or "penis" or "cervix mucus" or "semen" or "smegma" or "urine" or "vulva" or "vaginal discharge" or "urethral discharge" or "first*urine" or "urogenital discharge" or "urogenital secretion*" or "genital discharge" or "genital secretion*")
2.	ALL=("chlamydia*" or "chlamydia trachomatis" or "chlamydia* infection*")
3.	ALL=("antibod*" or "immunoglobulin*" or "T-cell*" or "B-cell*" or "helper*cell" or "cytokine*" or "interleukin*" or "interferon*" or "Treg*" or "Trm*" or "B-lymphocyte*" or "T-lymphocyte*" or "regulatory T*" or "tissue resident memory T*" or "adaptive immun*" or "innate immun*" or "mucosal immun*")
4.	#1 AND #2 AND #3
5.	#4 NOT TS=(filter described in reference 2)
**Scopus (Elsevier)**	
Search	Query
1.	TITLE-ABS-KEY ("meatal*" OR "penile*" OR "semen" OR "smegma" OR "urethra*" OR "cervicovaginal secretion*" OR "vagina*" OR "cervi*" OR "muc*" OR "urethritis" OR "urinary tract infection*" OR "penis" OR "cervix mucus" OR "semen" OR "smegma" OR "urine" OR "vulva" OR "vaginal discharge" OR "urethral discharge" OR "first*urine" OR "urogenital discharge" OR "urogenital secretion*" OR "genital discharge" OR "genital secretion*")
2.	TITLE-ABS-KEY ("chlamydia*" OR "chlamydia trachomatis" OR "chlamydia* infection*")
3.	TITLE-ABS-KEY ("antibod*" OR "immunoglobulin*" OR "T-cell*" OR "B-cell*" OR "helper*cell" OR "cytokine*" OR "interleukin*" OR "interferon*" OR "Treg*" OR "Trm*" OR "B-lymphocyte*" OR "T-lymphocyte*" OR "regulatory T*" OR "tissue resident memory T*" OR "adaptive immun*" OR "innate immun*" OR "mucosal immun*")
4.	#1 AND #2 AND #3
5.	#4 AND NOT ((INDEXTERMS (animals OR animal)) AND NOT (INDEXTERMS (humans OR human)) AND (LIMIT-TO (LANGUAGE , "English"))

Table S2. Risk of bias assessment of different studies included in this review.

Risk of bias assessment of analytical cross-sectional studies using the JBI critical appraisal tool. Y: yes, N: no, U: unclear, NA: not applicable. An overall appraisal is given. Questions were as follows: Q1: Were the criteria for inclusion in the sample clearly defined? Q2: Were the study subjects and the setting described in detail? Q3: Was the exposure measured in a valid and reliable way? Q4: Were objective, standard criteria used for measurement of the condition? Q5: Were confounding factors identified? Q6: Were strategies to deal with confounding factors stated? Q7: Were the outcomes measured in a valid and reliable way? Q8: Was appropriate statistical analysis used? For a detailed explanation, see reference [1].

First author, year[Ref.]	*Q1*	*Q2*	*Q3*	*Q4*	*Q5*	*Q6*	*Q7*	*Q8*	*Overall appraisal*
Albritton, 2017 [2]	Y	N	Y	Y	Y	Y	Y	Y	Some concerns
Ardizzone, 2021 [3]	Y	Y	Y	Y	N	Y	Y	Y	Some concerns
Brunham, 1983 [4]	Y	N	N	N	Y	Y	Y	Y	Some concerns
Cai, 2014 [5]	Y	Y	Y	Y	Y	Y	Y	Y	Low ROB
Agrawal, 2007 [6]	Y	Y	Y	Y	N	N	Y	Y	Some concerns
Darville, 2019 [7]	Y	Y	Y	Y	Y	Y	Y	Y	Low ROB
Bua, 2019 [8]	Y	Y	Y	N	N	Y	Y	Y	Some concerns
Agrawal, 2009 [9]	Y	Y	Y	Y	Y	Y	Y	Y	Low ROB
Cauci, 2007 [10]	Y	Y	Y	Y	U	N	Y	Y	Some concerns
Cohen, 1999 [11]	Y	N	Y	Y	Y	Y	Y	Y	Some concerns
Eggert-Kruse, 2011 [12]	Y	N	N	Y	Y	Y	Y	Y	Some concerns
Ogendi, 2017 [13]	Y	U	Y	Y	N	N	Y	Y	Some concerns
Martinez-Prado, 2010 [14]	Y	N	N	U	N	N	Y	Y	Some concerns
Ito, 2014 [15]	Y	Y	Y	U	Y	Y	Y	Y	Some concerns
Jha, 2011 [16]	Y	N	Y	Y	Y	Y	Y	Y	Some concerns
Mott, 2021 [17]	Y	Y	Y	Y	Y	Y	Y	Y	Low ROB
Lugo, 2023 [18]	Y	Y	Y	U	Y	Y	Y	Y	Low ROB
Ochsendorf, 1999 [19]	Y	Y	Y	Y	N	N	N	Y	Some concerns
Scott, 2006 [20]	Y	Y	N	Y	Y	Y	Y	Y	Some concerns
Spear, 2011 [21]	Y	N	Y	Y	Y	Y	Y	Y	Some concerns
Sperling, 2013 [22]	Y	Y	Y	Y	Y	Y	Y	Y	Low ROB
Fresse, 2010 [23]	Y	N	Y	N	N	N	Y	Y	High ROB
Perez-Soto, 2021 [24]	Y	Y	Y	Y	N	N	Y	Y	Some concerns
Kokab, 2010 [25]	Y	Y	Y	Y	N	N	Y	Y	Some concerns
Dehghan, 2016 [26]	Y	Y	Y	Y	N	N	Y	Y	Some concerns
Karaulov, 2017 [27]	N	N	N	N	Y	Y	Y	Y	High ROB
Witkin, 1997 [28]	N	N	Y	Y	Y	Y	N	NA	Some concerns
Perez-Soto, 2021 [29]	N	N	Y	Y	N	N	Y	Y	High ROB
Wolff, 1991 [30]	N	N	N	Y	Y	N	Y	Y	High ROB
Wolff, 1994 [31]	Y	Y	Y	Y	N	N	Y	Y	Some concerns
Witkin, 1996 [32]	Y	N	N	Y	U	Y	Y	Y	Some concerns
Witkin, 1994 [33]	Y	Y	Y	Y	N	U	Y	Y	Some concerns
Schust, 2012 [34]	N	N	Y	Y	U	N	Y	Y	High ROB
Mazzoli, 2010 [35]	Y	Y	Y	Y	U	N	Y	Y	Some concerns
Hakimi, 2014 [36]	N	N	Y	Y	U	N	Y	Y	High ROB
Mazzoli, 1996 [37]	Y	Y	Y	Y	Y	Y	N	Y	Some concerns
Bjercke, 1992 [38]	Y	N	N	U	N	N	N	NA	High ROB
Omer, 1985 [39]	N	N	N	U	Y	Y	Y	Y	Some concerns
Lewis, 2014 [40]	Y	N	Y	Y	Y	Y	Y	N	Some concerns
Shahmanesh, 1989 [41]	N	N	N	Y	N	N	N	Y	High ROB
Hammerschlag, 1978 [42]	N	N	N	Y	Y	Y	Y	N	Some concerns
Hammerschlag, 1979 [43]	N	N	N	Y	U	U	Y	Y	High ROB
Hakimi, 2014 [44]	Y	N	Y	Y	U	N	Y	Y	Some concerns
Habermann, 1999 [45]	Y	Y	N	Y	N	N	Y	Y	High ROB
McClure, 2013 [46]	Y	Y	Y	Y	Y	Y	U	Y	Some concerns
Moazenchi, 2017 [47]	Y	Y	Y	Y	N	N	N	Y	Some concerns
Ostaszewska-Puchalska, 2004 [48]	N	N	Y	Y	Y	U	N	Y	High ROB
Mahmoud, 1994 [49]	Y	Y	N	Y	N	N	Y	N	Some concerns
Chen, 2021 [50]	Y	Y	Y	Y	U	U	Y	Y	Some concerns
El-Din, 2021 [51]	Y	Y	Y	N	Y	Y	Y	Y	Some concerns
Richmond, 1980 [52]	N	N	N	Y	Y	Y	Y	U	Some concerns
Zhang, 2023 [53]	Y	Y	Y	Y	N	N	Y	Y	Some concerns
Gdoura, 2001 [54]	Y	N	Y	Y	Y	N	U	Y	Some concerns
Segnini, 2003 [55]	Y	N	N	N	N	N	Y	Y	High ROB
Ruijs, 1991 [56]	Y	Y	N	N	Y	Y	Y	Y	Some concerns
Motrich, 2006 [57]	Y	Y	Y	Y	Y	Y	Y	U	Some concerns
Penna, 2001 [58]	N	N	N	U	Y	Y	Y	Y	High ROB
Terho, 1981 [59]	N	N	N	N	N	N	Y	Y	High ROB
Marconi, 2014 [60]	Y	Y	Y	Y	U	U	Y	Y	Some concerns
McCormack, 1985 [61]	Y	Y	N	N	Y	Y	Y	Y	Some concerns
Jordan, 2017 [62]	Y	Y	Y	Y	Y	Y	Y	Y	Low ROB
Cai, 2011 [63]	Y	Y	Y	Y	Y	Y	N	Y	Some concerns

Ruijs, 1990 [64]	Y	N	N	Y	Y	Y	N	Y	Some concerns
Osser, 1984 [65]	Y	Y	N	U	N	N	Y	Y	Some concerns
Wiggins, 2006 [66]	Y	N	Y	Y	Y	Y	Y	Y	Some concerns
Gronroos, 1983 [67]	Y	Y	N	Y	Y	Y	Y	U	Some concerns
Darougar, 1981 [68]	Y	Y	N	Y	Y	Y	N	U	Some concerns
Dieterle, 1995 [69]	Y	N	Y	Y	N	N	N	Y	Some concerns
Eggert-Kruse, 1996 [70]	N	Y	N	Y	Y	Y	N	Y	Some concerns
Honkonen, 1983 [71]	N	N	N	Y	N	N	N	U	High ROB
Kojima, 1988 [72]	Y	Y	N	U	Y	Y	Y	Y	Some concerns
Mahmoud, 1995 [73]	Y	N	N	Y	Y	Y	U	Y	Some concerns
Mardh, 1978 [74]	Y	N	N	Y	N	Y	Y	Y	Some concerns
McComb, 1979 [75]	Y	Y	N	Y	U	Y	Y	Y	Some concerns
Munoz, 1996 [76]	Y	Y	N	U	N	N	U	Y	Some concerns
Schachter, 1979 [77]	N	N	N	NA	N	N	Y	Y	High ROB.
Munoz, 1995 [78]	Y	N	N	Y	N	N	Y	Y	Some concerns
Shahmanesh, 1996 [79]	N	N	N	Y	Y	Y	Y	Y	Some concerns
Thejls, 1995 [80]	Y	Y	N	NA	Y	Y	U	Y	Some concerns
Treharne, 1978 [81]	Y	N	N	U	Y	Y	Y	Y	Some concerns
Weidner, 1996 [82]	Y	N	N	Y	N	N	N	Y	Some concerns
Witkin, 1995 [83]	N	N	N	Y	Y	Y	N	Y	Some concerns
Yoshida, 1994 [84]	Y	N	N	Y	Y	Y	U	Y	Some concerns
Audu, 2025 [85]	N	N	N	Y	Y	Y	U	Y	Some concerns

Risk of bias assessment of case series using JBI critical appraisal tools. An overall appraisal is given. Y: yes, N: no, U: unclear, NA: not applicable. Questions were as follows: Q1: Were there clear criteria for inclusion in the case series? Q2: Was the condition measured in a standard, reliable way for all participants included in the case series? Q3: Were valid methods used for identification of the condition for all participants included in the case series? Q4: Did the case series have consecutive inclusion of participants? Q5: Did the case series have complete inclusion of participants? Q6: Was there clear reporting of the demographics of the participants in the study? Q7: Was there clear reporting of clinical information of the participants? Q8: Were the outcomes or follow-up results of cases clearly reported? Q9: Was there clear reporting of the presenting site(s)/clinic(s) demographic information? Q10: Was statistical analysis appropriate? For a detailed explanation see reference [86].

First author, year[Ref.]	*Q1*	*Q2*	*Q3*	*Q4*	*Q5*	*Q6*	*Q7*	*Q8*	*Q9*	*Q10*		*Overall appraisal*
Mardh, 1981 [87]	N	Y	Y	U	U	N	Y	Y	N	N		High ROB

Risk of bias assessment of text & opinion papers using JBI critical appraisal tools. An overall appraisal is given. Y: yes, N: no, U: unclear, NA: not applicable. Questions were as follows: Q1: Is the source of the opinion clearly identified? Q2: Does the source of opinion have standing in the field of expertise? Q3: Are the interests of the relevant population the central focus of the opinion? Q4: Is the stated position the result of an analytical process, and is there logic in the opinion expressed? Q5: Is there reference to the extant literature? Q6: Is any incongruence with the literature/sources logically defended? For a detailed explanation see reference [88].

First author, year[Ref.]	*Q1*	*Q2*	*Q3*	*Q4*	*Q5*	*Q6*		*Overall appraisal*
Ng, 1978 [89]	N	U	N	Y	N	U		High ROB

Risk of bias assessment of case reports using JBI critical appraisal tools. An overall appraisal is given. Y: yes, N: no, U: unclear, NA: not applicable. Questions were as follows: Q1: Were patient's demographic characteristics clearly described? Q2: Was the patient's history clearly described and presented as a timeline? Q3: Was the current clinical condition of the patient on presentation clearly described? Q4: Were diagnostic tests or methods and the results clearly described? Q5: Was the intervention(s) or treatment procedure(s) clearly described? Q6: Was the post-intervention clinical condition clearly described? Q7: Were adverse events (harms) or unanticipated events identified and described? Q8: Does the case report provide takeaway lessons? For a detailed explanation see reference [1]. Questions for text & opinion papers were as follows: Q1: Is the source of the opinion clearly identified? Q2. Does the source of opinion have standing in the field of expertise? Q3: Are the interests of the relevant population the central focus of the opinion? Q4: Is the stated position the result of an analytical process, and is there logic in the opinion expressed? Q5: Is there reference to the extant literature? Q6: Is any incongruence with the literature/sources logically defended? For a detailed explanation see reference [88].

First author, year[Ref.]	*Q1*	*Q2*	*Q3*	*Q4*	*Q5*	*Q6*	*Q7*		*Overall appraisal*
Gump, 1981 [90]	Y	Y	Y	Y	N	U	N		Some concerns
Ohsawa, 2000 [91]	N	Y	Y	Y	Y	U	Y		Some concerns

Risk of bias assessment of randomized controlled trials (RCT) using JBI critical appraisal tools. An overall appraisal is given. Y: yes, N: no, U: unclear, NA: not applicable. Questions were as follows: Q1: Was true randomization used for assignment of participants to treatment groups? Q2: Was allocation to groups concealed? Q3: Were treatment groups similar at the baseline? Q4: Were participants blind to treatment assignment? Q5: Were those delivering treatment blind to treatment assignment? Q6: Were outcomes assessors blind to treatment assignment? Q7: Were treatment groups treated identically other than the intervention of interest? Q8: Was follow up complete and if not, were differences between groups in terms of their follow up adequately described and analyzed? Q9: Were participants analyzed in the groups to which they were randomized? Q10 Were outcomes measured in the same way for treatment groups? Q11: Were outcomes measured in a reliable way? Q12: Was appropriate statistical analysis used? Q13: Was the trial design appropriate for the topic, and any deviations from the standard RCT design accounted for in the conduct and analysis? For a detailed explanation see reference [92].

First author, year[Ref.]	*Q1*	*Q2*	*Q3*	*Q4*	*Q5*	*Q6*	*Q7*	*Q8*	*Q9*	*Q10*	*Q11*	*Q12*	*Q13*	*Overall appraisal*
Cai, 2012 [93]	U	N	Y	N	N	Y	Y	Y	N	Y	Y	Y	Y	Some concerns
Abraham, 2019 [94]	Y	Y	Y	Y	N	Y	Y	Y	Y	Y	Y	Y	Y	Some concerns

Risk of bias assessment of case-control and cohort studies using the Newcastle-Ottawa scale. A total number of stars and overall appraisal is given. Questions for case-control studies were as follows: Q1: Is the case definition adequate? Q2: Representativeness of the cases. Q3: Selection of controls. Q4: Definition of controls. Q5: Comparability of cases and controls on the basis of the design or analysis. Q6: Ascertainment of exposure. Q7: Same method of ascertainment for cases and controls. Q8: Non-response rate. Questions for cohort studies were as follows: Q1: Representativeness of the exposed cohort. Q2: Selection of the non-exposed cohort. Q3: Ascertainment of exposure. Q4: Demonstration that outcome of interest was not present at start of the study. Q5: Comparability of cohorts on the basis of the design or analysis: study controls for age. Q6: Comparability of cohorts on the basis of design or analysis: study controls for any additional factor. Q7: Assessment of outcome. Q8: Was follow-up long enough for outcomes to occur? Q9: Adequacy of follow-up of cohorts. Further details on allocation of starts can be found in reference [95].

First author, year[Ref.]	*Study type*	*Q1*	*Q2*	*Q3*	*Q4*	*Q5*	*Q6*	*Q7*	*Q8*	*Q9*	*Total stars*	*Overall appraisal*
Ficarra, 2008 [96]	Cohort	*	NA	*	*	*	*	*	*		7	Some concerns
Wang, 2005a [97]	Cohort	*	NA	*		*	*	*	*	*	7	Some concerns
Mlisana, 2012 [98]	Cohort	*	*	*	*	*	*	*		*	8	Some concerns
Ziklo, 2018 [99]	Cohort	*	*	*	*			*	*		6	Some concerns
Ziklo, 2019 [100]	Cohort	*	*	*	*			*	*		6	Some concerns
Fichorova, 2020 [101]	Cohort	*	*	*	*	*		*	*	*	8	Some concerns
Poston, 2019 [102]	Cohort	*	NA	*		*	*	*	*	*	7	Some concerns
Hwang, 2015 [103]	Cohort	*	NA	*	*	*	*	*	*	*	8	Low ROB
Hedges, 1998 [104]	Cohort	*			*	*		*	*		5	Some concerns
Puolakkainen, 1986 [105]	Cohort	*	NA						*	*	3	High ROB
Workowski, 1993 [106]	Cohort	*	NA	*	*	*	*	*	*		7	Some concerns
Eggert-Kruse, 2001 [107]	Cohort	*	NA	*		*	*	*	NA	NA	5	Some concerns
Eggert-Kruse, 2007 [108]	Cohort	*	NA	*		*	*	*	NA	NA	5	Some concerns
Eggert-Kruse, 2002 [109]	Cohort	*	NA	*		*	*	*	NA	NA	5	Some concerns
Wang, 2005b [110]	Cohort	*	*	*	*			*	*	*	7	Some concerns
Southgate, 1983 [111]	Cohort		NA		*			*			2	High ROB
Garrett, 2021 [112]	Cohort	*	NA	*	*	*	*	*	*	*	8	Low ROB
Barousse, 2007 [113]	Cohort	*	*	*	*	*	*	*	*		8	Some concerns
Agrawal, 2007 [114]	Case-control	*	*	*		*	*	*			6	Some concerns
Agrawal, 2007 [115]	Case-control	*	*	*	*	*	*	*	*		8	Low ROB
Agrawal, 2008 [116]	Case-control	*	*	*		*	*	*			6	Some concerns
Agrawal, 2009 [117]	Case-control	*	*	*	*	*	*	*	*		8	Low risk of bias
Bollmann, 2001 [118]	Case-control	*	*								2	High risk of bias
Arno, 1990 [119]	Case-control	*	*					*			3	High risk of bias
Bollmann, 1998 [120]	Case-control				*				*		2	High risk of bias
Agrawal, 2011 [121]	Case-control	*	*	*	*	*	*	*	*		8	Low ROB
Agrawal, 2009 [122]	Case-control	*	*	*	*	*	*	*	*		8	Low ROB
Filardo, 2019 [123]	Case-control	*	*			*	*	*			5	Some concerns
Gupta, 2009 [124]	Case-control	*		*	*	*	*	*	*		7	Some concerns
Gupta, 2009 [125]	Case-control	*	*	*	*	*			*		6	Some concerns
Mazzoli, 2007 [126]	Case-control	*	*		*	*		*	*		6	Some concerns
Ibana, 2012 [127]	Case-control	*			*	*		*	*		5	Some concerns
Kelly, 2009 [128]	Case-control	*		*	*	*	*	*	*		7	Some concerns
Vats, 2007 [129]	Case-control	*		*	*	*		*	*		6	Some concerns
Srivastava, 2008 [130]	Case-control	*	*	*		*	*	*	*		7	Some concerns
Tsai, 2007 [131]	Case-control	*	*						*		3	High risk of bias
Fichorova, 2015 [132]	Case-control	*		*	*	*	*	*	*		7	Some concerns
Reddy, 2004 [133]	Case-control	*	*	*		*	*	*	*		7	Some concerns
Masson, 2015 [134]	Case-control			*	*	*	*	*	*		6	Some concerns
Markelova, 2016 [135]	Case-control					*			*		2	High ROB
Samra, 1994 [136]	Case-control		*	*	*	*			*		5	Some concerns
Persson, 1990 [137]	Case-control	*	*	*	*	*			*		6	Some concerns
El Feky, 2009 [138]	Case-control	*	*		*	*		*	*		6	Some concerns
Levine, 1998 [139]	Case-control	*	*	*	*		*		*		6	Some concerns
van den Broek, 2014 [140]	Case-control	*		*	*	*	*		*		6	Some concerns
Pate, 2001 [141]	Case-control	*	*	*	*			*	*		6	Some concerns
Jungwirth, 2003 [142]	Case-control								*		1	High ROB
Ezz-ElDIn, 2021 [143]	Case-control	*	*	*	*	*		*	*		7	Some concerns
Kalimo, 1981 [144]	Case-control		*		*	*			*		4	Some concerns
Suominen, 1983 [145]	Case-control	*			*				*		2	High ROB
Shortliffe, 1992 [146]	Case-control	*	*	*	*		*		*		6	Some concerns
Gruschwitz, 1996 [147]	Case-control					*	*		*		3	High ROB
McCormack, 1979 [148]	Case-control	*		*					*		3	High ROB
Ray, 2023 [149]	Case-control	*	*	*	*	*	*	*	*		8	Low ROB
Ray, 2024 [150]	Case-control	*	*	*	*	*	*	*	*		8	Low ROB

Table S6: Effect of most important influencing factors (coinfections, microbiome, hormonal effects, persistent or recurrent infections, and fertility disorders) on mucosal antibodies, cytokines, and immune cells.

		Mucosal antibodies	Mucosal cytokines	Mucosal immune cells
Coinfections	HPV	• No significant difference in CT sIgA positivity in ejaculate, EPS or PPM urine [5, a]	• Increased SP IL-1β, IL-6, IFN-γ [24, a]<br>• More CVL IL-1β fluctuations over time, less CVL RANTES fluctuations [103, a]	N/A
	NG	• Patients with previous NG showed higher risk of having high SP anti-Chlamydia IgA [58]	• No significant differences in IL-1β , IL-6, IL-8, IL-10, TFG-β concentrations in cervical mucus compared to NG alone [104]	• No significant differences in $CD4^+$ T-cells in endocervical brush [139, a]
	TV	N/A	N/A	• No significant differences in $CD4^+$ T-cells in endocervical brush [139, a]
	HSV-2	N/A	• Decreased ejaculate TNF-α, TNF-β, IL-10, IL-13 and increased IL-22 and IL-2sR, IFN-α, TGF-β [27, a]<br>• Increased ejaculate TNF-α compared to HSV-2 alone [135]	N/A
	MH/UU	• No significant difference anti-chlamydia LPS IgA [12]	N/A	N/A
Microbiome	Male	• Significantly increased *Proteus spp.* colonization in men with high anti-chlamydia LPS IgA in SP [12]<br>• No significant difference in anti-L2 IgA in EPS between those with or without bacterial prostatitis (with presence of *Streptococcus, Staphylococcus, Bacteroides, Clostridium spp.*) [146]	N/A	N/A
	Female (BV)	N/A	• No significant difference in IL-1β & IL-8 in vaginal fluid in BV+CT compared to BV [10]<br>• No significant difference in CVL IFN-γ in BV+CT [62, a]<br>• Lower IFN-α concentration in endocervical swabs of women with CST-I compared to CST-III and CST-IV. No significant difference in lactoferrin, IL-1α, IL-6, IFN-β and IFN-γ in CT+ women with different CST [123]<br>• CST-I is associated with IL-1α & IL-1β while CST-II and CST-III correlate with IL-6, IP-10 & RANTES in CVL [17]<br>• Increased CVL IL-1β, IL-6, and IL-8 in BV+CT compared to BV [60]<br>• Lower RANTES fluctuations in CVL over time between CT neg and CT pos follow-up [103]	• No significant difference in neutrophil numbers in vaginal smear in BV+CT compared to BV [10]<br>• No significant difference in PMN and T-cells in cervical cytobrushes [96, a]

Hormonal effect	Serum progesterone	N/A	• Negative correlation between CVL IL-1β and serum progesterone in women with recurrent infections [114]<br>• Negative correlation between IL-6, IFN-γ, TNF-α mRNA expression in urine and serum progesterone in CT+ women with recurrent spontaneous abortions [149]<br>• No significant association with cytokine levels (IL-4, IL-6, IL-8, IL-10, IL-12, TNF-α or IFN-γ) in CVL of fertile/FD women [116]	• No significant association with pDC or mDC count and CD80 expression in cervical cytobrush samples [116]
	Serum estradiol	N/A	• Decreased IL-10, IL-1β and IL-6 in CVL in women with primary infection [114]<br>• Increased IL-6 and IFN-γ in CVL in women with FD. No significant difference in other cytokines (IL-8, IL-10, IL-12, TNF-α) [116]	• Increased pDCs in cervical cytobrushes of women with FD and increased CD80 expression [116]
	Menstrual cycle	N/A	• No significant weekly variability over the course of the menstrual cycle in IFN-γ , IL-4, or IL-12 in cervical brush samples. IL-10 showed a n.s. trends towards higher levels around menstruation [20]	N/A
	Hormonal contraceptives	N/A	• No significant difference in CVL IFN-γ [62]<br>• Cervical swab β-defensin 2 and IL-1Rα ↓ in CT+ women who used DMPA compared to no HC. N.s. difference in other biomarkers (IL-1β, IL-6, IL-8, MIP-3α, VEGF, RANTES, ICAM-1, SLPI and IL-1RA/IL-1) [132]<br>• No significant difference in cervical swab IL-1β, IL-6, IL-8, MIP-3α, VEGF, RANTES, ICAM-1, IL-1RA, SLPI, BD-2 and IL-1RA/IL-1 between COC and no HC users [132]	N/A
Persistence & reinfection		• Decreased anti-MOMP IgG/IgA in CVL [115]<br>• Increased anti-cHsp IgG/IgA in CVL [115]<br>• Decreased anti-MOMP IgG in vaginal swab, while IgA did not differ significantly (STI clinic cohort) [140]<br>• Increased Anti-MOMP IgG/IgA in vaginal swab (fertility clinic cohort) [140]<br>• Increased IgG-secreting cervical cells in B-cell ELISpot *[115, c]*	• Increased IFN-γ in CVL [114,115] or non-significant increase [62]<br>• No significant difference in IL-1β, IL-6, or IL-10 in CVL [114,115]<br>• Increased IL-14, CXCL11, CXCL9, and CXCL10 in cervical sponge [102]<br>• Decreased IL-2 and increased IL-12 in endocervical sponge [97]<br>• Increased TGFβ1 mRNA expression in vaginal swabs [100]	• Increased lymphoproliferation of cervical cells to cHsp [115]<br>• Decreased lymphoproliferation of cervical cells to MOMP [115]<br>• Increased TLR2 expression on cervical monocytes [121]<br>• No significant difference in Foxp3 mRNA expression in vaginal swabs [100]

Fertility disorders	FD including MSA, subfertility or infertility	• Decreased anti-MOMP IgA in CVL [122]<br>• Increased cHsp-IgA in CVL [6,122]<br>• Increased cHsp-IgG in CVL [130]<br>• Increased anti-EB IgG, no significant difference in IgM in endocervical swab [85]	• Increased IFN-γ in CVL [6,116,122]<br>• Decreased IL-12 in CVL, no significant difference in TNF-α [116]<br>• Increased IL-6, IL-8 and IL-10 in CVL [116,122]<br>• Increased IL-1β in CVL [122]	• Decreased $CD4^+$ T-cells [116,117,122]<br>• Decreased monocytes[117], however, TLR2 is upregulated on these cells [121]<br>• Increased mDC and pDC, pDCs outnumber mDCs [122]<br>• Differential CD80 and CD83 expression on DCs [116].<br>• Increased IL-1β, IL-6, IL-8, IL-10, while IL-12 and IFN-γ are decreased in cervical cell supernatant [117, b]<br>• Increased IFN-γ, IL-10, and TNF-α, while no difference in IL-13 in cervical cell supernatant [130, c]<br>• Increased IL-4 and IL-10 SFC, while IFN-γ and IL-12 SFC decreased [117, b]<br>• Increased IL-4 and IL-10 mRNA expression in cervical cells [117, b]<br>• No significant difference in IL-1β, IL-6, IL-8, TNF-α mRNA expression in cervical cells [117, b]<br>• Decreased IFN-γ and IL-12 mRNA expression in cervical cells [117, b]
	Tubal pathology	• Increased vaginal swab anti-MOMP IgA, while no significant difference in IgG [140]	• Increased IFN-γ and IL-10, no significant difference in TNF-α and IL-6 in vaginal swab [50]	
	Worse pregnancy outcome	• Increased cHsp60-IgA in cervical swab [32]<br>• No significant difference in cervical swab anti-L2 IgA [67]<br>• No significant difference in IgA in cervical swab against pooled yolk sack antigens [65]<br>• Increased IgA against cHsp, MOMP and LPS in cervical swab medium [33]	N/A	N/A
	Male infertility	• No significant differences in anti-Chlamydial LPS IgA in SP [12]	N/A	N/A

BV: bacterial vaginosis, CST: community state type, CVL: cervicovaginal lavage, DMPA: injectable depot medroxyprogesterone acetate, EB: elementary bodies, FD: fertility disorders, HC: hormonal contraceptives, HPV: human papillomavirus, HSV-2: Herpes simplex virus-2, LPS: lipopolysaccharide, MH: Mycoplasma hominis, MOMP: major outer membrane protein, MSA: multiple spontaneous abortions, NG: Neisseria gonorrhoeae, pDC: plasmacytoid dendritic cells, PMN: polymorphonuclear cells, SP: seminal plasma, TV: Trichomonas vaginalis, UU: Ureaplasma urealyticum. [a]Compared to CT alone. [b]EB-stimulated cervical cells. [c]cHsp-stimulated cervical cells.

Figure S1: Number of studies investigating different cytokines and chemokines as indicated by length of the bar and annotated number. Different cytokines and chemokines are listed to the left. Cytokine family (colony-stimulating factors, interferons, interleukins, TNF-family) is indicating using colors. Studies measuring concentrations of hormones, enzymes, growth factors, antimicrobial peptides and proteins were not counted. IL-X: interleukine-X, IFN-X: interferon-X, TNF-α: tumor necrosis factor-α, CCL-X:CC chemokine (C-C motif) ligand-X, GM-CSF: granulocyte-macrophage colony-stimulating factor, G-CSF: granulocyte colony-stimulating factor, CXCL-X: chemokine (C-X-C motif) ligand-X, LT-α: lymphotoxin-α.

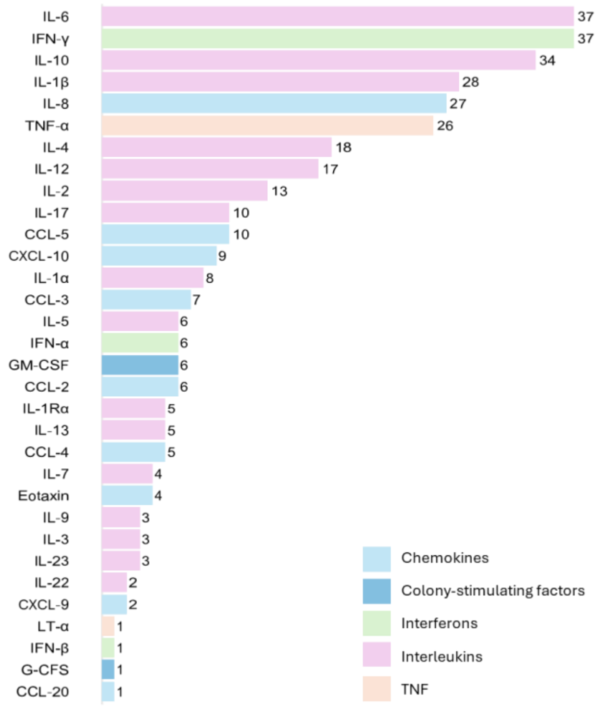

SUPPLEMENTAL REFERENCES

1. Moola S, M.Z., Tufanaru C, Aromataris E, Sears K, Sfetcu R, Currie M, Qureshi R, Mattis P, Lisy K, Mu P-F. (2020). Chapter 7: Systematic reviews of etiology and risk. In JBI Manual for Evidence Synthesis, M.Z. Aromataris E, ed.
2. Albritton, H.L., Kozlowski, P.A., Lillis, R.A., McGowin, C.L., Siren, J.D., Taylor, S.N., Ibana, J.A., Buckner, L.R., Shen, L., and Quayle, A.J. (2017). A novel whole-bacterial enzyme linked-immunosorbant assay to quantify Chlamydia trachomatis specific antibodies reveals distinct differences between systemic and genital compartments. Plos One *12*. ARTN e018310110.1371/journal.pone.0183101.
3. Ardizzone, C.M., Albritton, H.L., Lillis, R.A., Bagnetto, C.E.L., Shen, L., Cavacini, L.A., Kozlowski, P.A., and Quayle, A.J. (2021). Human genital antibody-mediated inhibition of Chlamydia trachomatis infection and evidence for ompA genotype-specific neutralization. Plos One *16*, e0258759. 10.1371/journal.pone.0258759.
4. Brunham, R.C., Kuo, C.C., Cles, L., and Holmes, K.K. (1983). Correlation of host immune response with quantitative recovery of Chlamydia trachomatis from the human endocervix. Infection and Immunity *39*, 1491-1494. 10.1128/iai.39.3.1491-1494.1983.
5. Cai, T., Wagenlehner, F.M.E., Mondaini, N., D'Elia, C., Meacci, F., Migno, S., Malossini, G., Mazzoli, S., and Bartoletti, R. (2014). Effect of human papillomavirus and Chlamydia trachomatis co-infection on sperm quality in young heterosexual men with chronic prostatitis-related symptoms. BJU International *113*, 281-287. 10.1111/bju.12244.
6. Agrawal, T., Vats, V., Salhan, S., and Mittal, A. (2007). Local markers for prediction of women at higher risk of developing sequelae to Chlamydia trachomatis infection. American Journal of Reproductive Immunology *57*, 153-159. 10.1111/j.1600-0897.2006.00459.x.
7. Darville, T., Albritton, H.L., Zhong, W., Dong, L., O'Connell, C.M., Poston, T.B., Quayle, A.J., Goonetilleke, N., Wiesenfeld, H.C., Hillier, S.L., and Zheng, X. (2019). Anti-chlamydia IgG and

IgA are insufficient to prevent endometrial chlamydia infection in women, and increased anti-chlamydia IgG is associated with enhanced risk for incident infection. American Journal of Reproductive Immunology *81*, e13103. 10.1111/aji.13103.
8. Bua, A., Cannas, S., Zanetti, S., and Molicotti, P. (2019). Levels of different cytokines in women and men with asymptomatic genital infection caused by Chlamydia. Journal of infection in developing countries *13*, 847-850. 10.3855/jidc.9810.
9. Agrawal, T., Vats, V., Wallace, P.K., Singh, A., Salhan, S., and Mittal, A. (2009). Recruitment of myeloid and plasmacytoid dendritic cells in cervical mucosa during Chlamydia trachomatis infection. Clinical Microbiology and Infection *15*, 50-59. 10.1111/j.1469-0691.2008.02113.x.
10. Cauci, S., and Culhane, J.F. (2007). Modulation of vaginal immune response among pregnant women with bacterial vaginosis by Trichomonas vaginalis, Chlamydia trachomatis, Neisseria gonorrhoeae, and yeast. American Journal of Obstetrics and Gynecology *196*, 133.e131-133.e137. 10.1016/j.ajog.2006.08.033.
11. Cohen, C.R., Plummer, F.A., Mugo, N., Maclean, I., Shen, C., Bukusi, E.A., Irungu, E., Sinei, S., Bwayo, J., and Brunham, R.C. (1999). Increased interleukin-10 in the endocervical secretions of women with non-ulcerative sexually transmitted diseases: A mechanism for enhanced HIV-1 transmission? AIDS *13*, 327-332. 10.1097/00002030-199902250-00004.
12. Eggert-Kruse, W., Weltin, M., and Strowitzki, T. (2011). Are Chlamydial Lipopolysaccachide-directed Antibodies in Seminal Plasma or Serum Clinically Significant During Investigation of Male Infertility? UROLOGY *77*, 1101-1106. 10.1016/j.urology.2010.11.014.
13. Ogendi, B.M.O., Bakshi, R.K., Sabbaj, S., Brown, L., Lee, J.Y., Kapil, R., and Geisler, W.M. (2017). Distinct peripheral vs mucosal T-cell phenotypes in chlamydia-infected women. American Journal of Reproductive Immunology *78*. ARTN e1276810.1111/aji.12768.
14. Martínez-Prado, E., and Camejo Bermúdez, M.I. (2010). Expression of IL-6, IL-8, TNF-α, IL-10, HSP-60, Anti-HSP-60 Antibodies, and Anti-sperm Antibodies, in Semen of Men with Leukocytes and/or Bacteria. AMERICAN JOURNAL OF REPRODUCTIVE IMMUNOLOGY *63*, 233-243. 10.1111/j.1600-0897.2009.00786.x.
15. Ito, S., Horie, K., Seike, K., Yasuda, M., Tsuchiya, T., Yokoi, S., Nakano, M., and Deguchi, T. (2014). Usefulness of quantifying leukocytes in first-voided urine to predict positivity for Chlamydia trachomatis in asymptomatic men at high risk for chlamydial infection. Journal of infection and chemotherapy : official journal of the Japan Society of Chemotherapy *20*, 748-751. https://dx.doi.org/10.1016/j.jiac.2014.08.002.
16. Jha, R., Srivastava, P., Salhan, S., Finckh, A., Gabay, C., Mittal, A., and Bas, S. (2011). Spontaneous secretion of interleukin-17 and -22 by human cervical cells in Chlamydia trachomatis infection. Microbes and Infection *13*, 167-178. 10.1016/j.micinf.2010.10.012.
17. Mott, P.D., Taylor, C.M., Lillis, R.A., Ardizzone, C.M., Albritton, H.L., Luo, M., Calabresi, K.G., Martin, D.H., Myers, L., and Quayle, A.J. (2021). Differences in the Genital Microbiota in Women Who Naturally Clear Chlamydia trachomatis Infection Compared to Women Who Do Not Clear; A Pilot Study. Frontiers in Cellular and Infection Microbiology *11*, 615770. 10.3389/fcimb.2021.615770.
18. Lugo, L.Z.A., Puga, M.A.M., Jacob, C.M.B., Padovani, C.T.J., Nocetti, M.C., Tupiná, M.S., Pina, A.F.S., de Freitas, J.N.M., Ferreira, A.M.T., dos Santos Fernandes, C.E., et al. (2023). Cytokine profiling of samples positive for Chlamydia trachomatis and Human papillomavirus. Plos One *18*, e0279390. 10.1371/journal.pone.0279390.
19. Ochsendorf, F.R., Özdemir, K., Rabenau, H., Fenner, T., Oremek, R., Milbradt, R., and Doerr, H.W. (1999). Chlamydia trachomatis and male infertility: Chlamydia-IgA antibodies in seminal plasma are C. trachomatis specific and associated with an inflammatory response. Journal of the European Academy of Dermatology and Venereology *12*, 143-152. 10.1016/S0926-9959(98)00150-0.
20. Scott, M.E., Ma, Y., Farhat, S., Shiboski, S., and Moscicki, A.-B. (2006). Covariates of cervical cytokine mRNA expression by real-time PCR in adolescents and young women: effects of Chlamydia trachomatis infection, hormonal contraception, and smoking. Journal of clinical immunology *26*, 222-232.
21. Spear, G.T., Kendrick, S.R., Chen, H.Y., Thomas, T.T., Bahk, M., Balderas, R., Ghosh, S., Weinberg, A., and ay, A.L. (2011). Multiplex immunoassay of lower genital tract mucosal fluid from women attending an urban STD clinic shows broadly increased IL1s and lactoferrin. Plos One *6*, e19560. doi:https://dx.doi.org/10.1371/journal.pone.0019560.
22. Sperling, R., Kraus, T.A., Ding, J., Veretennikova, A., Lorde-Rollins, E., Singh, T., Lo, Y., Quayle, A.J., and Chang, T.L. (2013). Differential profiles of immune mediators and in vitro HIV infectivity between endocervical and vaginal secretions from women with Chlamydia trachomatis

infection: A pilot study. Journal of Reproductive Immunology *99*, 80-87. 10.1016/j.jri.2013.07.003.
23. Fresse, A.S., Sueur, J.M., and Hamdad, F. (2010). Diagnosis and follow-up of genital chlamydial infection by direct methods and by detection of serum IgG, IgA and secretory IgA. INDIAN JOURNAL OF MEDICAL MICROBIOLOGY *28*, 326-331. 10.4103/0255-0857.71823.
24. Pérez-Soto, E., Fernández-Martínez, E., Oros-Pantoja, R., Medel-Flores, O., Mir, a-Covarrubias, J.C., and Sánchez-Monroy, V. (2021). Proinflammatory and oxidative stress states induced by human papillomavirus and chlamydia trachomatis coinfection affect sperm quality in asymptomatic infertile men. Medicina (Lithuania) *57*. doi:10.3390/medicina57090862.
25. Kokab, A., Akhondi, M.M., Sadeghi, M.R., Modarresi, M.H., Aarabi, M., Jennings, R., Pacey, A.A., and Eley, A. (2010). Raised Inflammatory Markers in Semen From Men With Asymptomatic Chlamydial Infection. JOURNAL OF ANDROLOGY *31*, 114-120. 10.2164/jandrol.109.008300.
26. Dehghan Marvast, L., Aflatoonian, A., Talebi, A.R., Ghasemzadeh, J., and Pacey, A.A. (2016). Semen inflammatory markers and Chlamydia trachomatis infection in male partners of infertile couples. Andrologia *48*, 729-736. 10.1111/and.12501.
27. Karaulov, A.V., Markelova, E.V., Chepurnova, N.S., Tulupova, M.S., Nevezhkina, T.A., Hamoshina, M.B., Zhdanova, O.L., Mizanova, V.Z., and Ivanjuk, N.A. (2017). Cytokine profile and intracellular matrix component degradation indicators in male ejaculate with chronic urethritis before and following the treatment. Asian Journal of Pharmaceutics *11*, S865-S872.
28. Witkin, S.S., Bongiovanni, A.M., and Inglis, S.R. (1997). Detection of endocervical anti-Chlamydia trachomatis immunoglobulin A in pregnant women by a rapid, 6-minute enzyme-linked immunosorbent assay: Comparison with PCR and chlamydial antigen detection methods. JOURNAL OF CLINICAL MICROBIOLOGY *35*, 1781-1783. 10.1128/JCM.35.7.1781-1783.1997.
29. Pérez-Soto, E., Oros-Pantoja, R., Fernández-Martínez, E., Carbonell-Campos, J.M., and Monroy, V.S. (2021). Seminal pro-inflammatory cytokines and pH are affected by Chlamydia infection in asymptomatic patients with teratozoospermia. Central European Journal of Immunology *46*, 76-81. 10.5114/ceji.2021.105247.
30. Wolff, H., Neubert, U., Zebhauser, M., Bezold, G., Korting, H.C., and Meurer, M. (1991). Chlamydia trachomatis induces an inflammatory response in the male genital tract and is associated with altered semen quality. Fertility and Sterility *55*, 1017-1019. 10.1016/S0015-0282(16)54318-0.
31. Wolff, H., Neubert, U., Volken, t, M., Zochling, N., Schlupen, E.M., Bezold, G., and Meurer, M. (1994). Detection of Chlamydia trachomatis in semen by antibody-enzyme immunoassay compared with polymerase chain reaction, antigen-enzyme immunoassay, and urethral cell culture. Fertility and Sterility *62*, 1250-1254. doi:10.1016/s0015-0282(16)57194-5.
32. Witkin, S.S., Jeremias, J., Neuer, A., David, S., Kligman, I., Toth, M., Willner, E., and Witkin, K. (1996). Immune Recognition of the 60kD Heat Shock Protein: Implications for Subsequent Fertility. Infectious Diseases in Obstetrics and Gynecology *4*, 152-158. 10.1155/S1064744996000336.
33. Witkin, S.S., Sultan, K.M., Neal, G.S., Jeremias, J., Grifo, J.A., and Rosenwaks, Z. (1994). Unsuspected Chlamydia trachomatis infection and in vitro fertilization outcome. American Journal of Obstetrics and Gynecology *171*, 1208-1214. 10.1016/0002-9378(94)90134-1.
34. Schust, D.J., Ibana, J.A., Buckner, L.R., Ficarra, M., Sugimoto, J., Amedee, A.M., and Quayle, A.J. (2012). Potential mechanisms for increased HIV-1 transmission across the endocervical epithelium during C. trachomatis infection. Current HIV Research *10*, 218-227. 10.2174/157016212800618093.
35. Mazzoli, S., Cai, T., Addonisio, P., Bechi, A., Mondaini, N., and Bartoletti, R. (2010). Chlamydia trachomatis Infection Is Related to Poor Semen Quality in Young Prostatitis Patients. European Urology *57*, 708-714. 10.1016/j.eururo.2009.05.015.
36. Hakimi, H., Zainodini, N., Khorramdelazad, H., Arababadi, M.K., and Hassanshahi, G. (2014). Seminal levels of pro-inflammatory (CXCL1, CXCL9, CXCL10) and homeostatic (CXCL12) chemokines in men with asymptomatic Chlamydia trachomatis infection. Jundishapur Journal of Microbiology *7*, e11152. 10.5812/jjm.11152.
37. Mazzoli, S., Meacci, F., Cosco, E., and Poggiali, C. (1996). Clinical consequences of immune responses to Chlamydia in men. Infect Dis Obstet Gynecol *4*, 136-142. 10.1155/S1064744996000300.
38. Bjercke, S., and Purvis, K. (1992). Chlamydial serology in the investigation of infertility. Hum Reprod *7*, 621-624. 10.1093/oxfordjournals.humrep.a137707.

39. Omer, E.F.E., Forsey, T., Darougar, S., Ali, M.H., and el-Naeem, H.A. (1985). Seroepidemiological survey of chlamydial genital infections in Khartoum, Sudan. Genitourinary Medicine *61*, 261-263. 10.1136/sti.61.4.261.
40. Lewis, M.E., Belland, R.J., AbdelRahman, Y.M., Beatty, W.L., Aiyar, A.A., Zea, A.H., Greene, S.J., Marrero, L., Buckner, L.R., Tate, D.J., et al. (2014). Morphologic and molecular evaluation of Chlamydia trachomatis growth in human endocervix reveals distinct growth patterns. Frontiers in Cellular and Infection Microbiology *4*, 71. 10.3389/fcimb.2014.00071.
41. Shahmanesh, M. (1989). Characteristics of inflammatory cells in urethral smears from men with non-gonococcal urethritis. Genitourinary medicine *65*, 18-21.
42. Hammerschlag, M.R., Alpert, S., Rosner, I., Thurston, P., Semine, D., McComb, D., and McCormack, W.M. (1978). Microbiology of the vagina in children: Normal and potentially pathogenic organisms. Pediatrics *62*, 57-62.
43. Hammerschlag, M.R., Anderka, M., Semine, D.Z., McComb, D., and McCormack, W.M. (1979). Prospective study of maternal and infantile infection with Chlamydia trachomatis. Pediatrics *64*, 142-148.
44. Hakimi, H., Akhondi, M.M., Sadeghi, M.R., Chamani, L., Arababadi, M.K., Ahmadabadi, B.N., Hassanshahi, G., and Fathollahi, M.S. (2014). Seminal Levels of IL-10, IL-12, and IL-17 in Men with Asymptomatic Chlamydia Infection. INFLAMMATION *37*, 122-126. 10.1007/s10753-013-9719-7.
45. Habermann, B., and Krause, W. (1999). Altered sperm function or sperm antibodies are not associated with chlamydial antibodies in infertile men with leucocytospermia. JOURNAL OF THE EUROPEAN ACADEMY OF DERMATOLOGY AND VENEREOLOGY *12*, 25-29. 10.1111/j.1468-3083.1999.tb00803.x.
46. McClure, C.P., Bowman, C.A., Geary, I., Ryan, C., Ball, J.K., and Eley, A. (2013). HIV-1 co-receptor expression and epithelial immune cells of the cervix in asymptomatic women attending a genitourinary medicine clinic. HIV MEDICINE *14*, 108-114. 10.1111/hiv.12002.
47. Moazenchi, M., Totonchi, M., Salman Yazdi, R., Hratian, K., Mohseni Meybodi, M.A., Ahmadi Panah, M., Chehrazi, M., and Mohseni Meybodi, A. (2018). The impact of Chlamydia trachomatis infection on sperm parameters and male fertility: A comprehensive study. International Journal of STD and AIDS *29*, 466-473. 10.1177/0956462417735245.
48. Ostaszewska-Puchalska, I., Zdrodowska-Stefanow, B., Badyda, J., Bulhak-Koziol, V., Pucilo, K., and Darewicz, B. (2004). Antichlamydial antibodies in the serum and expressed prostatic secretion in prostatitis. ARCHIVUM IMMUNOLOGIAE ET THERAPIAE EXPERIMENTALIS *52*, 277-283.
49. Mahmoud, E.A., Hamad, E.E., Bassiri, M., and Mardh, P.A. (1994). Antichlamydial activity of cervical secretion. Journal of Obstetrics and Gynaecology *14*, 166-171. 10.3109/01443619409004068.
50. Chen, H., Wang, L., Zhao, L., Luo, L., Min, S., Wen, Y., Lei, W., Shu, M., and Li, Z. (2021). Alterations of Vaginal Microbiota in Women With Infertility and Chlamydia trachomatis Infection. Frontiers in Cellular and Infection Microbiology *11*, 698840. 10.3389/fcimb.2021.698840.
51. El-din, A.N., Sorour, H., Fattouh, M., and Abu El-Hamd, M. (2021). Evaluation of the role of <i>Chlamydia trachomatis</i> in primary male infertility. INTERNATIONAL JOURNAL OF CLINICAL PRACTICE, e14702. 10.1111/ijcp.14702.
52. Richmond, S.J., Milne, D.J., Hilton, A.L., and Caul, E.O. (1980). Antibodies to chlamydia trachomatis in cervicovaginal secretions: Relation to serum antibodies and current chlamydial infection. Sexually Transmitted Diseases *7*, 11-15. 10.1097/00007435-198001000-00003.
53. Zhang, S., Funahashi, Y., Tanaka, S., Okubo, T., Thapa, J., Nakamura, S., Higashi, H., and Yamaguchi, H. (2023). Chlamydia trachomatis relies on the scavenger role of aryl hydrocarbon receptor with detyrosinated tubulin for its intracellular growth, but this is impaired by excess indole. Microbes and Infection *25*, 105097. 10.1016/j.micinf.2022.105097.
54. Gdoura, R., Daoudi, F., Bouzid, F., Ben Salah, F., Chaigneau, C., Sueur, J.M., Eb, F., Rekik, S., Hammami, A., and Orfila, J. (2001). Detection of Chlamydia trachomatis in semen and urethral specimens from male members of infertile couples in Tunisia. The European journal of contraception & reproductive health care : the official journal of the European Society of Contraception *6*, 14-20.
55. Segnini, A., Camejo, M.I., and Proverbio, F. (2003). Chlamydia trachomatis and sperm lipid peroxidation in infertile men. Asian Journal of Andrology *5*, 47-49.
56. Ruijs, G.J., Kauer, F.M., Jager, S., Schroder, F.P., Schirm, J., and Kremer, J. (1991). Further details on sequelae at the cervical and tubal level of Chlamydia trachomatis infection in infertile women. Fertility and Sterility *56*, 20-26. 10.1016/s0015-0282(16)54410-0.

57. Motrich, R.D., Cuffini, C., Mackern Oberti, J.P., Maccioni, M., and Rivero, V.E. (2006). Chlamydia trachomatis occurrence and its impact on sperm quality in chronic prostatitis patients. Journal of Infection *53*, 175-183. 10.1016/j.jinf.2005.11.007.
58. Penna Videau, S., Cermeno Vivas, J., and Salazar, N. (2001). IgA antibodies to Chlamydia trachomatis and seminal parameters in asymptomatic infertile males. Archives of andrology *46*, 189-195.
59. Terho, P., and Meurman, O. (1981). Chlamydial serum IgG, IgA and local IgA antibodies in patients with genital-tract infections measured by solid-phase radioimmunoassay. Journal of Medical Microbiology *14*, 77-87. 10.1099/00222615-14-1-77.
60. Marconi, C., Santos-Greatti, M.M.V., Parada, C., Pontes, A., Pontes, A.G., Giraldo, P.C., Donders, G.G.G., and da Silva, M.G. (2014). Cervicovaginal Levels of Proinflammatory Cytokines Are Increased During Chlamydial Infection in Bacterial Vaginosis But Not in Lactobacilli-Dominated Flora. JOURNAL OF LOWER GENITAL TRACT DISEASE *18*, 261-265.
61. McCormack, W.M., Rosner, B., McComb, D.E., Evrard, J.R., and Zinner, S.H. (1985). Infection with Chlamydia trachomatis in female college students. American Journal of Epidemiology *121*, 107-115. 10.1093/oxfordjournals.aje.a113971.
62. Jordan, S.J., Olson, K.M., Barnes, S., Wilson, L.S., Berryhill, T.F., Bakshi, R., Brown, L.T., Press, C.G., and Geisler, W.M. (2017). Lower Levels of Cervicovaginal Tryptophan Are Associated With Natural Clearance of Chlamydia in Women. J Infect Dis *215*, 1888-1892. 10.1093/infdis/jix240.
63. Cai, T., Mondaini, N., Migno, S., Meacci, F., Boddi, V., Gontero, P., Malossini, G., Geppetti, P., Mazzoli, S., and Bartoletti, R. (2011). Genital Chlamydia trachomatis Infection is Related to Poor Sexual Quality of Life in Young Sexually Active Women. Journal of Sexual Medicine *8*, 1131-1137. 10.1111/j.1743-6109.2010.02194.x.
64. Ruijs, G.J., Kauer, F.M., Jager, S., Schroder, P.F., Schirm, J., and Kremer, J. (1990). Is serology of any use when searching for correlations between Chlamydia trachomatis infection and male infertility? Fertility and Sterility *53*, 131-136. 10.1016/s0015-0282(16)53228-2.
65. Osser, S., and Persson, K. (1984). Postabortal pelvic infection associated with Chlamydia trachomatis and the influence of humoral immunity. American Journal of Obstetrics and Gynecology *150*, 699-703. 10.1016/0002-9378(84)90670-7.
66. Wiggins, R.C., Holmes, C.H., Andersson, M., Ibrahim, F., Low, N., and Horner, P.J. (2006). Quantifying leukocytes in first catch urine provides new insights into our understanding of symptomatic and asymptomatic urethritis. INTERNATIONAL JOURNAL OF STD & AIDS *17*, 289-295. 10.1258/095646206776790268.
67. GrÖNroos, M., Honkonen, E., Terho, P., and Punnonen, R. (1983). Cervical and serum IgA and serum IgG antibodies to Chlamydia trachomatis and herpes simplex virus in threatened abortion: a prospective study. BJOG: An International Journal of Obstetrics & Gynaecology *90*, 167-170. 10.1111/j.1471-0528.1983.tb08903.x.
68. Darougar, S., Forsey, T., Wood, J.J., Bolton, J.P., and Allan, A. (1981). Chlamydia and the Curtis-Fitz-Hugh syndrome. British Journal of Venereal Diseases *57*, 391-394. 10.1136/sti.57.6.391.
69. Dieterle, S., Mahony, J.B., Luinstra, K.E., and Stibbe, W. (1995). Immunology: Chlamydial immunoglobulin IgG and IgA antibodies in serum and semen are not associated with the presence of chlamydia trachomatis DNA or rRNA in semen from male partners of infertile couples. Human Reproduction *10*, 315-319. 10.1093/oxfordjournals.humrep.a135934.
70. Eggert-Kruse, W., Buhlinger-Göpfarth, N., Rohr, G., Probst, S., Aufenanger, J., Näher, H., and Runnebaum, B. (1996). Antibodies to Chlamydia trachomatis in semen and relationship with parameters of male fertility. Human Reproduction *11*, 1408-1417. 10.1093/oxfordjournals.humrep.a019410.
71. Honkonen, E., Punnonen, R., and Terho, P. (1983). Chlamydia trachomatis in term pregnancy. Isolation and serological study with a case report. International Journal of Gynecology and Obstetrics *21*, 473-476. 10.1016/0020-7292(83)90037-1.
72. Kojima, H., Wang, S.P., Kuo, C.C., and Grayston, J.T. (1988). Local antibody in semen for rapid diagnosis of Chlamydia trachomatis epididymitis. Journal of Urology *140*, 528-531. 10.1016/S0022-5347(17)41710-1.
73. Mahmoud, E.A., Svensson, L.O., Olsson, S.E., and Mårdh, P.A. (1995). Antichlamydial activity of vaginal secretion. American Journal of Obstetrics and Gynecology *172*, 1268-1272. 10.1016/0002-9378(95)91491-9.
74. Mardh, P.A., Ripa, K.T., Colleen, S., Treharne, J.D., and Darougar, S. (1978). Role of Chlamydia trachomatis in non-acute prostatitis. British Journal of Venereal Diseases *54*, 330-334. 10.1136/sti.54.5.330.

75. McComb, D.E., Nichols, R.L., Semine, D.Z., Evrard, J.R., Alpert, S., Crockett, V.A., Rosner, B., Zinner, S.H., and McCormack, W.M. (1979). Chlamydia trachomatis in women: Antibody in cervical secretions as a possible indicator of genital infection. J Infect Dis *139*, 628-633. 10.1093/infdis/139.6.628.
76. Munoz, M.G., Jeremias, J., and Witkin, S.S. (1996). The 60 kDa heat shock protein in human semen: Relationship with antibodies to spermatozoa and Chlamydia trachomatis. Human Reproduction *11*, 2600-2603. 10.1093/oxfordjournals.humrep.a019177.
77. Schachter, J., Cles, L., Ray, R., and Hines, P.A. (1979). Failure of serology in diagnosing chlamydial infections of the female genital tract. Journal of Clinical Microbiology *10*, 647-649. 10.1128/jcm.10.5.647-649.1979.
78. Munoz, M.G., and Witkin, S.S. (1995). Immunology: Autoimmunity to spermatozoa, asymptomatic chlamydia trachomatis genital tract infection and γδ t lymphocytes in seminal fluid from the male partners of couples with unexplained infertility. Human Reproduction *10*, 1070-1074. 10.1093/oxfordjournals.humrep.a136096.
79. Shahmanesh, M., it, P.G., and Round, R. (1996). Urethral lymphocyte isolation in non-gonococcal urethritis. Genitourinary Medicine *72*, 362-364. doi:10.1136/sti.72.5.362.
80. Thejls, H., Rahm, V.A., Gnarpe, J., and Gnarpe, H. (1995). Diagnostic efficacy of chlamydial antibodies in cervical secretions from pregnant women and adolescent girls. Genitourinary Medicine *71*, 370-374. 10.1136/sti.71.6.370.
81. Treharne, J.D., Darougar, S., Simmons, P.D., and Thin, R.N. (1978). Rapid diagnosis of chlamydial infection of the cervix. British Journal of Venereal Diseases *54*, 403-408. 10.1136/sti.54.6.403.
82. Weidner, W., Floren, E., Zimmermann, O., Thiele, D., and Ludwig, M. (1996). Chlamydial antibodies in semen: Search for "silent" chlamydial infections in asymptomatic andrological patients. INFECTION *24*, 309-313. 10.1007/BF01743366.
83. Witkin, S.S., Kligman, I., and Bongiovanni, A.M. (1995). Relationship between an asymptomatic male genital tract exposure to Chlamydia trachomatis and an autoimmune response to spermatozoa. HUMAN REPRODUCTION *10*, 2952-2955. 10.1093/oxfordjournals.humrep.a135827.
84. Yoshida, K., Kobayashi, N., and Negishi, T. (1994). Chlamydia trachomatis infection in the semen of asymptomatic infertile men: detection of the antigen by in situ hybridization. Urologia internationalis *53*, 217-221.
85. Audu, O., Musa, B.O.P., Usman, A., Adekunle, O.O., Opaluwa, S.A., El-Fulaty, A.A., Olayemi, B., Okwubenata, O.L., Ega, B., Yaqub, Y., and Oraebosi, M.I. (2025). Comparative assessment of Chlamydia trachomatis pathogen prevalence, and the determination of host gyneco-epidemiological and immunological associated risk factors in female infertility. Cytokine *185*, 156819. https://dx.doi.org/10.1016/j.cyto.2024.156819.
86. Munn Z, B.T., Moola S, Tufanaru C, Stern C, McArthur A, Stephenson M, Aromataris, and E. Methodological quality of case series studies. JBI Evidence Synthesis. doi: 10.11124/JBISRIR-D-19-00099.
87. Mardh, P.A., Moller, B.R., and Ingerselv, H.J. (1981). Endometritis caused by Chlamydia trachomatis. British Journal of Venereal Diseases *57*, 191-195.
88. McArthur A, K.J., Yan H, Florescu S. (2015). Innovations in the systematic review of text and opinion. . Int J Evid Based Healthc. *13*, 188-195.
89. Ng, K.M., Graham, D.M., Forsyth, J.R., and Brennan, K.F. (1978). Antichlamydial antibody in genital exudates of men and women with non-gonococcal genital infections. Lancet (London, England) *1*, 507.
90. Gump, D.W., Dickstein, S., and Gibson, M. (1981). Endometritis related to Chlamydia trachomatis infection. Annals of Internal Medicine *95*, 61-63. 10.7326/0003-4819-95-1-61.
91. Ohsawa, I., Ohi, H., Endo, M., Fujita, T., Hidaka, M., Satomura, A., and Yamaguchi, Y. (2001). A case of renal involvement in persistent immune activation caused by chlamydial salpingitis. Virchows Archiv *438*, 306-311. 10.1007/s004280000314.
92. Tufanaru C, M.Z., Aromataris E, Campbell J, Hopp L. (2020). Chapter 3: Systematic reviews of effectiveness. In JBI Manual for Evidence Synthesis. , M.Z. Aromataris E, ed.
93. Cai, T., Wagenlehner, F.M.E., Mazzoli, S., Meacci, F., Mondaini, N., Nesi, G., Tiscione, D., Malossini, G., and Bartoletti, R. (2012). Semen quality in patients with Chlamydia trachomatis genital infection treated concurrently with prulifloxacin and a phytotherapeutic agent. Journal of Andrology *33*, 615-623. 10.2164/jandrol.111.013961.
94. Abraham, S., Juel, H.B., Bang, P., Cheeseman, H.M., Dohn, R.B., Cole, T., Kristiansen, M.P., Korsholm, K.S., Lewis, D., Olsen, A.W., et al. (2019). Safety and immunogenicity of the

chlamydia vaccine candidate CTH522 adjuvanted with CAF01 liposomes or aluminium hydroxide: a first-in-human, randomised, double-blind, placebo-controlled, phase 1 trial. The Lancet. Infectious diseases *19*, 1091-1100. https://dx.doi.org/10.1016/S1473-3099(19)30279-8.

95. Wells, G.A., Wells, G., Shea, B., Shea, B., O'Connell, D., Peterson, J., Welch, Losos, M., Tugwell, P., Ga, S.W., et al. (2014). The Newcastle-Ottawa Scale (NOS) for Assessing the Quality of Nonrandomised Studies in Meta-Analyses.
96. Ficarra, M., Ibana, J.S.A., Poretta, C., Ma, L., Myers, L., Taylor, S.N., Greene, S., Smith, B., Hagensee, M., Martin, D.H., and Quayle, A.J. (2008). A Distinct Cellular Profile Is Seen in The Human Endocervix During Chlamydia trachomatis Infection. American Journal of Reproductive Immunology *60*, 415-425. 10.1111/j.1600-0897.2008.00639.x.
97. Wang, C., Tang, J., Crowley-Nowick, P.A., Wilson, C.M., Kaslow, R.A., and Geisler, W.M. (2005). Interleukin (IL)-2 and IL-12 responses to Chlamydia trachomatis infection in adolescents. Clinical and Experimental Immunology *142*, 548-554. 10.1111/j.1365-2249.2005.02946.x.
98. Mlisana, K., Naicker, N., Werner, L., Roberts, L., Van Loggerenberg, F., Baxter, C., Passmore, J.A.S., Grobler, A.C., Sturm, A.W., Williamson, C., et al. (2012). Symptomatic vaginal discharge is a poor predictor of sexually transmitted infections and genital tract inflammation in high-risk women in South Africa. J Infect Dis *206*, 6-14. 10.1093/infdis/jis298.
99. Ziklo, N., Vidgen, M.E., Taing, K., Huston, W.M., and Timms, P. (2018). Dysbiosis of the vaginal microbiota and higher vaginal kynurenine/tryptophan ratio reveals an association with Chlamydia trachomatis genital infections. Frontiers in Cellular and Infection Microbiology *8*, 1. 10.3389/fcimb.2018.00001.
100. Ziklo, N., Huston, W.M., Taing, K., and Timms, P. (2019). High expression of IDO1 and TGF-beta1 during recurrence and post infection clearance with Chlamydia trachomatis, are independent of host IFN-gamma response. BMC infectious diseases *19*, 218. https://dx.doi.org/10.1186/s12879-019-3843-4.
101. Fichorova, R.N., Morrison, C.S., Chen, P.-L., Yamamoto, H.S., Govender, Y., Junaid, D., Ryan, S., Kwok, C., Chipato, T., Salata, R.A., and Doncel, G.F. (2020). Aberrant cervical innate immunity predicts onset of dysbiosis and sexually transmitted infections in women of reproductive age. Plos One *15*, e0224359. https://dx.doi.org/10.1371/journal.pone.0224359.
102. Poston, T.B., Lee, D.A.E., Darville, T., Zhong, W., Dong, L., O'Connell, C.M., Wiesenfeld, H.C., Hillier, S.L., Sempowski, G.D., and Zheng, X. (2019). Cervical Cytokines Associated With Chlamydia trachomatis Susceptibility and Protection. The Journal of infectious diseases *220*, 330-339. https://dx.doi.org/10.1093/infdis/jiz087.
103. Hwang, L.Y., Scott, M.E., Ma, Y., and Moscicki, A.-B. (2015). Diversity of Cervicovaginal Cytokine Response to Incident Chlamydia trachomatis Infection Among a Prospective Cohort of Young Women. American journal of reproductive immunology (New York, N.Y. : 1989) *74*, 228-236. https://dx.doi.org/10.1111/aji.12401.
104. Hedges, S.R., Sibley, D.A., Mayo, M.S., Hook, E.W., 3rd, and Russell, M.W. (1998). Cytokine and antibody responses in women infected with Neisseria gonorrhoeae: effects of concomitant infections. The Journal of infectious diseases *178*, 742-751.
105. Puolakkainen, M., Vesterinen, E., Purola, E., Saikku, P., and Paavonen, J. (1986). Persistence of chlamydial antibodies after pelvic inflammatory disease. Journal of Clinical Microbiology *23*, 924-928. 10.1128/jcm.23.5.924-928.1986.
106. Workowski, K.A., Lampe, M.F., Wong, K.G., Watts, M.B., and Stamm, W.E. (1993). Long-term Eradication of Chlamydia trachomatis Genital Infection After Antimicrobial Therapy: Evidence Against Persistent Infection. JAMA: The Journal of the American Medical Association *270*, 2071-2075. 10.1001/jama.1993.03510170061031.
107. Eggert-Kruse, W., Boit, R., Rohr, G., Aufenanger, J., Hund, M., and Strowitzki, T. (2001). Relationship of seminal plasma interleukin (IL)-8 and IL-6 with semen quality. HUMAN REPRODUCTION *16*, 517-528. 10.1093/humrep/16.3.517.
108. Eggert-Kruse, W., Kiefer, I., Beck, C., Demirakca, T., and Strowitzki, T. (2007). Role for tumor necrosis factor alpha (TNF-α) and interleukin 1-beta (IL-1β) determination in seminal plasma during infertility investigation. FERTILITY AND STERILITY *87*, 810-823. 10.1016/j.fertnstert.2006.08.103.
109. Eggert-Kruse, W., Neuer, A., Clussmann, C., Boit, R., Geissler, W., Rohr, G., and Strowitzki, T. (2002). Seminal antibodies to human 60kd heat shock protein (HSP 60) in male partners of subfertile couples. HUMAN REPRODUCTION *17*, 726-735. 10.1093/humrep/17.3.726.
110. Wang, C., Tang, J., Geisler, W.M., Crowley-Nowick, P.A., Wilson, C.M., and Kaslow, R.A. (2005). Human leukocyte antigen and cytokine gene variants as predictors of recurrent Chlamydia trachomatis infection in high-risk adolescents. J Infect Dis *191*, 1084-1092. 10.1086/428592.

111. Southgate, L.J., Treharne, J.D., and Forsey, T. (1983). Chlamydia trachomatis and Neisseria gonorrhoeae infections in women attending inner city general practices. Br Med J (Clin Res Ed) *287*, 879-881. 10.1136/bmj.287.6396.879.
112. Garrett, N., Mtshali, A., Osman, F., Masson, L., McKinnon, L.R., Singh, R., Mitchev, N., Ngobese, H., Kharsany, A.B.M., Abdool Karim, S., et al. (2021). Impact of point-of-care testing and treatment of sexually transmitted infections and bacterial vaginosis on genital tract inflammatory cytokines in a cohort of young South African women. Sexually transmitted infections *97*, 555-565. https://dx.doi.org/10.1136/sextrans-2020-054740.
113. Barousse, M.M., Theall, K.P., Van Der Pol, B., Fortenberry, J.D., Orr, D.P., and Fidel, P.L. (2007). Susceptibility of middle adolescent females to sexually transmitted infections: Impact of hormone contraception and sexual behaviors on vaginal immunity. American Journal of Reproductive Immunology *58*, 159-168. 10.1111/j.1600-0897.2007.00504.x.
114. Agrawal, T., Vats, V., Wallace, P.K., Salhan, S., and Mittal, A. (2007). Cervical cytokine responses in women with primary or recurrent chlamydial infection. JOURNAL OF INTERFERON AND CYTOKINE RESEARCH *27*, 221-226. 10.1089/jir.2006.0132.
115. Agrawal, T., Vats, V., Salhan, S., and Mittal, A. (2007). Mucosal and peripheral immune responses to chlamydial heat shock proteins in women infected with Chlamydia trachomatis. Clinical and Experimental Immunology *148*, 461-468. 10.1111/j.1365-2249.2007.03357.x.
116. Agrawal, T., Vats, V., Wallace, P.K., Salhan, S., and Mittal, A. (2008). Role of cervical dendritic cell subsets, co-stimulatory molecules, cytokine secretion profile and beta-estradiol in development of sequalae to Chlamydia trachomatis infection. REPRODUCTIVE BIOLOGY AND ENDOCRINOLOGY *6*, 46. 10.1186/1477-7827-6-46.
117. Agrawal, T., Gupta, R., Dutta, R., Srivastava, P., Bhengraj, A.R., Salhan, S., and Mittal, A. (2009). Protective or pathogenic immune response to genital chlamydial infection in women-A possible role of cytokine secretion profile of cervical mucosal cells. Clinical Immunology *130*, 347-354. 10.1016/j.clim.2008.10.004.
118. Bollmann, R., Engel, S., Petzoldt, R., and Göbel, U.B. (2001). Chlamydia trachomatis in andrologic patients - Direct and indirect detection. Infection *29*, 113-118. 10.1007/s15010-001-0088-z.
119. Arno, J.N., Ricker, V.A., Batteiger, B.E., Katz, B.P., Caine, V.A., and Jones, R.B. (1990). Interferon-gamma in endocervical secretions of women infected with Chlamydia trachomatis. The Journal of infectious diseases *162*, 1385-1389.
120. Bollmann, R., Engel, S., Sagert, D., and Gobel, U.B. (1998). Investigations on the detection of Chlamydia trachomatis infections in infertile male outpatients. ANDROLOGIA *30*, 23-27.
121. Agrawal, T., Bhengraj, A.R., Vats, V., Salhan, S., and Mittal, A. (2011). Expression of TLR 2, TLR 4 and iNOS in Cervical Monocytes of Chlamydia trachomatis-infected Women and Their Role in Host Immune Response. American Journal of Reproductive Immunology *66*, 534-543. 10.1111/j.1600-0897.2011.01064.x.
122. Agrawal, T., Vats, V., Salhan, S., and Mittal, A. (2009). Determination of chlamydial load and immune parameters in asymptomatic, symptomatic and infertile women. FEMS IMMUNOLOGY AND MEDICAL MICROBIOLOGY *55*, 250-257. 10.1111/j.1574-695X.2008.00530.x.
123. Filardo, S., Di Pietro, M., Tranquilli, G., Latino, M.A., Recine, N., Porpora, M.G., and Sessa, R. (2019). Selected Immunological Mediators and Cervical Microbial Signatures in Women with Chlamydia trachomatis Infection. mSystems *4*. https://dx.doi.org/10.1128/mSystems.00094-19.
124. Gupta, R., Srivastava, P., Vardhan, H., Salhan, S., and Mittal, A. (2009). Host immune responses to chlamydial inclusion membrane proteins B and C in Chlamydia trachomatis infected women with or without fertility disorders. REPRODUCTIVE BIOLOGY AND ENDOCRINOLOGY *7*, 38. 10.1186/1477-7827-7-38.
125. Gupta, R., Vardhan, H., Srivastava, P., Salhan, S., and Mittal, A. (2009). Modulation of cytokines and transcription factors (T-Bet and GATA3) in CD4 enriched cervical cells of Chlamydia trachomatis infected fertile and infertile women upon stimulation with chlamydial inclusion membrane proteins B and C. REPRODUCTIVE BIOLOGY AND ENDOCRINOLOGY *7*, 84. 10.1186/1477-7827-7-84.
126. Mazzoli, S., Cai, T., Rupealta, V., Gavazzi, A., Castricchi Pagliai, R., Mondaini, N., and Bartoletti, R. (2007). Interleukin 8 and Anti-Chlamydia trachomatis Mucosal IgA as Urogenital Immunologic Markers in Patients with C. trachomatis Prostatic Infection. European Urology *51*, 1385-1393. 10.1016/j.eururo.2006.10.059.
127. Ibana, J.A., Myers, L., Porretta, C., Lewis, M., Taylor, S.N., Martin, D.H., and Quayle, A.J. (2012). The major CD8 T cell effector memory subset in the normal and Chlamydia trachomatis-infected human endocervix is low in perforin. BMC Immunology *13*, 66. 10.1186/1471-2172-13-66.

128. Kelly, K.A., Wiley, D., Wiesmeier, E., Briskin, M., Butch, A., and Darville, T. (2009). The combination of the gastrointestinal integrin (α4β7) and selectin ligand enhances T-cell migration to the reproductive tract during infection with chlamydia trachomatis. pp. 446-452.
129. Vats, V., Agrawal, T., Salhan, S., and Mittal, A. (2007). Primary and secondary immune responses of mucosal and peripheral lymphocytes during Chlamydia trachomatis infection. FEMS Immunology and Medical Microbiology *49*, 280-287. 10.1111/j.1574-695X.2006.00196.x.
130. Srivastava, P., Jha, R., Bas, S., Salhan, S., and Mittal, A. (2008). In infertile women, cells from Chlamydia trachomatis infected site release higher levels of interferon-gamma, interleukin-10 and tumor necrosis factor-alpha upon heat shock protein stimulation than fertile women. Reproductive Biology and Endocrinology *6*, 20. 10.1186/1477-7827-6-20.
131. Tsai, P.Y., Hsu, M.C., Huang, C.T., and Li, S.Y. (2007). Human antibody and antigen response to IncA antibody of Chlamydia trachomatis. International Journal of Immunopathology and Pharmacology *20*, 155-161. 10.1177/039463200702000118.
132. Fichorova, R.N., Chen, P.-L., Morrison, C.S., Doncel, G.F., Mendonca, K., Kwok, C., Chipato, T., Salata, R., and Mauck, C. (2015). The Contribution of Cervicovaginal Infections to the Immunomodulatory Effects of Hormonal Contraception. mBio *6*, e00221-00215. https://dx.doi.org/10.1128/mBio.00221-15.
133. Reddy, B.S., Rastogi, S., Das, B., Salhan, S., Verma, S., and Mittal, A. (2004). Cytokine expression pattern in the genital tract of Chlamydia trachomatis positive infertile women - Implication for T-cell responses. Clinical and Experimental Immunology *137*, 552-558. 10.1111/j.1365-2249.2004.02564.x.
134. Masson, L., Salkinder, A.L., Olivier, A.J., McKinnon, L.R., Gamieldien, H., Mlisana, K., Scriba, T.J., Lewis, D.A., Little, F., Jaspan, H.B., et al. (2015). Relationship between female genital tract infections, mucosal interleukin-17 production and local T helper type 17 cells. Immunology *146*, 557-567. https://dx.doi.org/10.1111/imm.12527.
135. Markelova, E.V., Chepurnova, N.S., Tulupova, M.S., Karaulov, A.V., Baibarina, E.V., Mel'nikova, Y.A., Shadrina, M.N., Yakovleva, Y., Rabota, N.S., and Ziangirova, Y.V. (2016). Study of the system of cytokines in case of herpetic and chlamydia-herpetic infection. Journal of Global Pharma Technology *8*, 10-14.
136. Samra, Z., Soffer, Y., and Pansky, M. (1994). Prevalence of genital chlamydia and mycoplasma infection in couples attending a male infertility clinic. European Journal of Epidemiology *10*, 69-73. 10.1007/BF01717455.
137. Persson, E., Eneroth, P., and Grillner, L. (1990). Immunoglobulin contents in cervical secretions of women with chlamydial cervicitis. Gynecologic and Obstetric Investigation *30*, 109-113. 10.1159/000293229.
138. El Feky, M.A., Hassan, E.A., El Din, A.M., Hofny, E.R., Afifi, N.A., Eldin, S.S., and Baker, M.O. (2009). Chlamydia trachomatis: methods of identification and impact on semen quality. The Egyptian journal of immunology / Egyptian Association of Immunologists *16*, 49-59.
139. Levine, W.C., Pope, V., Bhoomkar, A., Tambe, P., Lewis, J.S., Zaidi, A.A., Farshy, C.E., Mitchell, S., and Talkington, D.F. (1998). Increase in endocervical CD4 lymphocytes among women with nonulcerative sexually transmitted diseases. The Journal of infectious diseases *177*, 167-174.
140. van den Broek, I.V.F., , J., A., e., van Bergen, J.E.A.M., Morre, S.A., S, v.d., and e, M.A.B. (2014). Chlamydia trachomatis Antibody Testing in Vaginal Mucosal Material versus Blood Samples of Women Attending a Fertility Clinic and an STI Clinic. Obstetrics and gynecology international *2014*, 601932. doi:https://dx.doi.org/10.1155/2014/601932.
141. Pate, M.S., Hedges, S.R., Sibley, D.A., Russell, M.W., Hook, E.W., 3rd, and Mestecky, J. (2001). Urethral cytokine and immune responses in Chlamydia trachomatis-infected males. Infection and immunity *69*, 7178-7181.
142. Jungwirth, A., Straberger, A., Esterbauer, B., Fink, K., and Schmeller, N. (2003). Acrosome reaction in Chlamydia-positive and negative patients. pp. 314-316.
143. EzzEl-Din, A.M., Gaber, H.D., and Kamal, D.T. (2021). Chlamydia trachomatis Infection: its relation to semen parameters and sperm DNA integrity. The Egyptian journal of immunology *28*, 290-298.
144. Kalimo, K., Terho, P., Honkonen, E., Gronroos, M., and Halonen, P. (1981). Chlamydia trachomatis and herpes simplex virus IgA antibodies in cervical secretions of patients with cervical atypia. British journal of obstetrics and gynaecology *88*, 1130-1134.
145. Suominen, J., Grönroos, M., Terho, P., and Wichmann, L. (1983). Chronic prostatitis, chlamydia trachomatis and infertility. International Journal of Andrology *6*, 405-413. 10.1111/j.1365-2605.1983.tb00555.x.

146. Shortliffe, L.M.D., Sellers, R.G., and Schachter, J. (1992). THE CHARACTERIZATION OF NONBACTERIAL PROSTATITIS - SEARCH FOR AN ETIOLOGY. JOURNAL OF UROLOGY *148*, 1461-1466.
147. Gruschwitz, M.S., Brezinschek, R., and Brezinschek, H.P. (1996). Cytokine levels in the seminal plasma of infertile males. Journal of Andrology *17*, 158-163.
148. McCormack, W.M., Alpert, S., McComb, D.E., Nichols, R.L., Semine, D.Z., and Zinner, S.H. (1979). Fifteen-month follow-up study of women infected with Chlamydia trachomatis. N Engl J Med *300*, 123-125. 10.1056/NEJM197901183000305.
149. Ray, A., Bhati, T., Arora, R., and Rastogi, S. (2023). Progesterone-mediated immunoregulation of cytokine signaling by miRNA-133a and 101-3p in Chlamydia trachomatis-associated recurrent spontaneous abortion. Molecular Immunology *164*, 47-57. 10.1016/j.molimm.2023.10.012.
150. Ray, A., Pradhan, D., Siraj, F., Arora, R., and Rastogi, S. (2024). MicroRNA mediated regulation of oxidative stress and cytokines in Chlamydia trachomatis-infected recurrent spontaneous abortion: A case-control study. American Journal of Reproductive Immunology *91*, e13821. 10.1111/aji.13821.
